# Supplementary material for: A modality-independent proto-organization of human multisensory areas
Source: Nat Hum Behav. 2023 Jan 16;7(3):397–410. doi: 10.1038/s41562-022-01507-3 (PMC10038796; doi:10.1038/s41562-022-01507-3)
Supplement: Supplementary file 1 — Supplementary discussion, methods, Figs. 1–6 and Tables 1–3. [file 41562_2022_1507_MOESM1_ESM.docx]

**Table of Contents**

Supplementary Results…………...………………...page 2

Supplementary Discussion……….………………...page 3

Supplementary Methods………….………………...page 9

Supplementary Fig.1…………...……………………page 19

Supplementary Fig.2…………...……………………page 21

Supplementary Fig.3…………...……………………page 22

Supplementary Fig.4…………...……………………page 24

Supplementary Fig.5…………...……………………page 26

Supplementary Fig.6…………...……………………page 27

Supplementary Table1…………...………………….page 28

Supplementary Table2…………...………………….page 31

Supplementary Table3…………...………………….page 32

References……………………………………………page 38

**Supplementary Results**

**Behavioral assessment**

Familiarity with the movie plot was assessed through a Likert scale ranging from 1 to 5. The majority of the participants reported to have a general knowledge of the main facts of the narrative (TD median: 3; SD median 2,5; overall four subjects declared no familiarity at all with the movie, whereas none of them stated to know the story very well).

After the scanning session, an *ad hoc* two-alternative forced choice questionnaire about the content of the story was administered to assess subjects’ engagement and compliance. In the first two experimental sessions, all TD participants attended the movie as resulted from our assessment questionnaire (N = 30, mean accuracy ± standard deviation = 87% ± 7%; range_min-max_ = 72 - 100%). Similarly, in the third experiment, all SD participants attended the movie as resulted from the final questionnaire (N = 18, mean accuracy ± standard deviation = 82% ± 13%; range_min-max_ = 56% - 100%).

**Supplementary Discussion**

**The posterior cingulate cortex is synchronized across meaningful auditory and visual movie**

Results of the ISC analysis showed a shared recruitment of the posterior cingulate cortex (a hub of the Default Mode Network -DMN-) across the two unimodal conditions of stimulus presentation (i.e., A *vs* V) that may be associated with high-level cognitive processes related to narrative understanding.

Such finding is compatible with previous evidence reporting a common engagement of the DMN and the STS/STG, during the encoding of naturalistic information^1,2^, processing of spoken and written natural language^3^ and comprehension of naturalistic narrative speech^4-6^. This hypothesis is further supported by the findings of the current study. First, the results of the model-mediated ISC revealed that the shared processing of visual and auditory streams in the posterior cingulate cortex is mainly driven by high-level features associated to the movie semantics (i.e., linguistic and category-related information). Second, the synchronization across auditory and visual streams in this region is disrupted by the presentation of a scrambled movie condition, in which the chronological order of cuts is purposely altered to make the storyline nonsensical. To this regard, while synchronization of brain activity among viewers does occur in a network of brain areas comprising the posterior cingulate cortex for the processing of both coherent silent videos and meaningful auditory movies, this same synchronization is instead dampened by the presentation of random/scrambled visual sequences or meaningless audio clips^7^. Finally, TRW analysis demonstrated that the temporal dynamics of input processing in this region are relatively slow (of the order of minutes), thus reflecting a high-level stage of information processing, compatible with a role of the posterior cingulate cortex in story understanding. Taken together, these observations suggest that the posterior cingulate cortex may represent the stimulus narrative^3^ in which meaningful events are accumulated and integrated over relatively long timescales^8,9^.

**The inferior frontal cortex is not synchronized across SD samples**

Integration of signals across modalities relies both on object knowledge and previous experience in relating sensory properties belonging to the same stimulus^10^. Such process counts on the recruitment of a fronto-temporal network comprising perisylvian temporal and inferior frontal regions, prefrontal cortex and the precuneus^5^ which are involved in object recognition^11^, comprehension of non-linguistic conceptual information^12^, discourse understanding and working-memory^13,14^. Specifically, the left inferior frontal gyrus is engaged during the processing of everyday, bimodal audiovisual stimulation, thus providing a modality-independent neural substrate crucial for relating information that is shared across the two senses^15^.

For what concerns congenital blindness and deafness, semantic knowledge is acquired both via a sensory-grounded event representation dependent on the spared senses^16,17^, and through high-level, sensory independent mechanisms based on linguistic description and cognitive inference^18,19^ and relying on a distributed network of regions comprising the frontal cortex. Additionally, large-scale modifications in the functional coupling among brain areas has been demonstrated in congenitally blind individuals which present an enhanced functional connectivity between the primary visual cortex and the inferior frontal gyrus/sulcus^10,20,21^.

Moreover, ISC analysis revealed congenitally blind individuals commonly engage the inferior frontal gyrus/sulcus and middle frontal junction while listening to auditory movies^7^. Similarly, a recruitment of the left inferior/middle frontal gyri has been found in congenitally deaf signers during the presentation of isolated written English words and pictorial stimuli^22^. Therefore, we expected to find a significant ISC in the inferior frontal gyrus across visual and auditory movie processing in both TD and SD samples. However, we found that only in TD participants the left inferior frontal gyrus and the bilateral medial prefrontal cortex were synchronized across auditory and visual conditions, while synchronization across deaf and blind participants did not occur. Several explanations can account for this finding. To begin with, this lack of synchronization may depend on the differences in language processing between the two groups of congenitally deprived participants, whose post-natal sensory experiences inevitably differ. Indeed, the absence of specific sensory experiences early in life affects the development and the (left) lateralization of the neural systems devoted to language processing: while an overlapping left-lateralized, fronto-temporal circuitry is engaged during the processing of information in the native language (for a meta-analytic review see^23^) both for native signers and hearing individuals during speech, when asked to read written sentences, native signers do not show activation in these areas^24^. Therefore, given this evidence and considering the properties of our stimulus that requires subtitles reading, we can speculate that our native signers minimally recruited the left frontal regions while watching the movie. Analogously, congenital loss of visual input in blindness affects language processing and determines a reduced left-lateralization of language functions^25,26^. Consistently, we previously showed that the left IFG was not included in cortical regions involved in semantic processing across sighted and congenitally blind individuals while performing a property-generation task with concrete nouns, presented through visual and/or auditory modalities^18^. Differences in the neural correlates of naturalistic information processing across sensory deprived groups could indeed emerge as a result of differences in visually- and acoustically- based semantic representation of events. In fact, evidence demonstrated that the left inferior frontal gyrus is involved in semantic processing of sentences^27,28^ and is sensitive to the semantic congruence across stimuli, as conveyed by different sensory modalities such as pictures-sounds^29^ and actions-language (gestures and pantomimes) combinations^30^. Moreover, evidence exists of a disconnection between sensory regions and higher order areas in sensory deprivation^31^ and this may possibly explain the lack of synchronization of IFG cortex across blind and deaf individuals. Thus, left IFG participates in the online matching of information across two sensory streams (e.g., gestures with the accompanying speech) to build a novel, context-dependent semantic representation of the external events^32^. Given these considerations, we can assume that the lack of multisensory experience in coupling speech sounds with the related visual gestures in both deaf and blind participants may prevent the creation of common, shared representations of the same sensory events.

**Movie editing drives ISC across conditions**

A movie results from the editing of quick shots into scenes: artificial sequences that retain the unity of time and location through an accurate mapping of the auditory soundscape with the visual stream. Such a technical process determines the pace of the audiovisual stimulation and the final rhythm of the narrative, ultimately creating a definite stream of meaningful sequences (i.e., scene transitions and camera cuts) that convey the story. Since synchronization of brain activity in fMRI mainly arose from slow-frequency fluctuations^33^, we explored the structure of the movie in order to model what we called the “movie editing” features. Therefore, with this term, we refer to both the editor choices (e.g., scenes, camera cuts, dialogues, soundtracks) and the modifications we made, namely the inclusion of the audiodescriptions and subtitles.

Note that the movie editing model, by the way it is conceptualized, can be conceived as a ‘blanket’ term, encompassing a set of non-specific, visual, auditory and linguistic stimulus properties that cannot be unequivocally assigned to any of the other stimulus models we included in our analysis pipeline. In fact, changes in the movie scenery, for instance, likely result in modifications of the visual properties of the image, often accompanied by variations in the soundscape. Therefore, not surprisingly, it shared a consistent portion of variance in the data with all the other computational models we included in our analysis (Supplementary Fig.2), thus representing an ideal covariate to control for slow temporal changes which might be modulated by other brain processes (e.g., working memory, attentional mechanisms, change in arousal, etc.) apart from sensory processing^34^.

Results of the encoding of the editing model (Supplementary Fig.2), revealed that these features significantly impacted brain synchronization across the whole cortex, ranging from the visual and auditory regions, as well as ventral and dorsal attention networks in the frontal and parietal lobes^35^, and the language network^36^. Moreover, the editing model was tested as a mediator during ISC (Supplementary Fig.4) in the A *vs* V experimental conditions. Results showed that half of the ISC magnitude in STS/STG was driven by this set of features.

These observations suggest that the movie editing descriptor captures some slow-changing stimulus characteristics, mediated both by bottom-up and top-down (e.g., attentional, arousal and memory) modulations. Such hypothesis follows from the idea that movie understanding requires concurrent deployment of several perceptual and cognitive processes necessary for the creation of a meaningful and coherent representation of the information impinging our senses.

**Limitations**

Our study presents the following specificities and limitations.

First, since the aim of the present study was to assess whether the emergence of audiovisual processing in the human brain requires audiovisual experience, the work mainly focused on the study of synchronized brain responses across experimental groups and conditions. Therefore, the investigation of group-specific responses or experience-dependent reorganization phenomena that take place when a sensory modality is absent goes beyond the scope of the current study.

Second, the small sample size (N = 18) of sensory deprived groups could be considered as limited. However, a review of the current literature in the field of sensory deprivation reveals that many studies rely on a comparable number of subjects (e.g.,^37-39^) mainly for the difficulties related to the recruitment of these exceptional participants who lack sensory input since birth. For what concerns the field of naturalistic stimulation and ISC analysis, although some studies report bigger sample sizes, they do often rely on the presentation of shorter clips/audio descriptions relative to the long movie (~1h) stimulation adopted here. For this reason, the data of the present work are still aligned with the average number of datapoints reported in the literature (for further details on this please see Supplementary Fig.6 and Supplementary Table 3).

Third, we acknowledge that deaf participants were not matched for age to the other experimental groups, being on average, younger than the other samples (i.e., deaf: N = 9, mean age 24 ± 4 years; blind: N = 9, mean age 44 ± 14 years; TD: AV condition, N = 10, 35 ± 13 years; N = 10, A condition, 39 ± 17 years; V condition, N = 10, 37 ± 15 years). Even if this discrepancy is mainly related to the exceptionality of congenitally deprived samples and in the difficulty of their recruitment, which does not allow a ‘selection’ based on age, a parsimonious evaluation of this age gaps between samples could not justify the differences that has been reported between groups. In addition, here, deaf individuals have been primarily analyzed for their commonalities with the blind group and the TD samples.

In addition, we have to mention that subtitles and lip-movement in the visual movie are not fully congruent, since they rely on Italian for subtitles and English for acting, as commonly occurs in Italy for dubbed movies. Nevertheless, we believe that the presence of subtitles hardly allowed participants to concurrently perform lip-reading. Furthermore, this discrepancy would solely affect the V-only version of the movie for both TD and deaf samples, and -in case- would negatively affect the overlapping responses across samples. Since we are yet demonstrating significant overlapping responses across samples, this point represents an issue of way lesser concern.

Moreover, we would like to mention that one issue might be represented by the low temporal resolution of fMRI that, due to the hemodynamic response time constraints, does not allow a precise characterization of brain responses occurring at fine-grained temporal scales (i.e., fast signal fluctuations). However, although fMRI has not the same temporal resolution of electrophysiological methods (e.g., ECoG, EEG or MEG), several examples do exist that show how BOLD signal could still be exploited to get information about the temporal properties of visual^40^ and acoustic (e.g., speech and sound processing^41-43^) stimuli. In the present work we adopted an analytical approach called Temporal Receptive Window (TRW) analysis that has been widely used in the fMRI field^13,34,44,45^ to characterize the temporal structure of information processing during naturalistic stimulation. Although the temporal information in our study is not comparable to a frequency-domain analysis (as done in electrophysiology) to quantify ongoing temporal coupling between neural activity and stimulus properties, the information we got from the coarse organization of temporal tunings represented into the TRW maps (see Fig.5) may be a useful and additional piece of evidence to characterize the properties of multisensory regions.

Finally, it is important to highlight that a large portion of the common variance, as extracted by the movie editing descriptor, has been initially removed from the models in the mediation analysis (see Methods and Supplementary Fig.2b). This procedure aimed at better characterizing the contributions of high- and low-level feature models in explaining common brain activity (i.e., ISC), even if dealing with a lower explained variance. Indeed, the set of coarse features, included in the movie editing descriptor, can be described by any other high- or low-level model (Supplementary Fig.2b), thus undermining the discussion on the specific contribution of each of these computational models in the mediation process. As a matter of fact, the movie editing descriptor alone accounted for about half of the intersubject correlation (e.g., in A *vs* V in TD participants, ISC in the peak in left STS passed from r ~ 0.214 to r ~ 0.127 with an effect of ~ 0.087 when using the movie editing as a mediator), as reported in Supplementary Fig.3. Moreover, the magnitude of the model-mediated effect of the movie editing features was on average four-fold as compared to the other models (e.g., in A *vs* V in TD participants, mediation effect for the low-level model in left STS was r ~ 0.018). The small effect sizes of the computational models were generated by the orthogonalization procedure with the movie editing descriptors. When removing the movie editing features from the computational models, model-mediated ISC accounted on average for one third of the original magnitude (e.g., in A *vs* V in TD participants, mediation for the low-level model in left STS passed from r ~ 0.105 to r ~ 0.017 when moving from the original descriptors to the cleaned ones). Although the small effect size of the cleaned models had a significant impact in mediation of ISC, one could consider this effect as negligible. Conversely, we would like to emphasize that:

1. the orthogonalization procedure was intended to favor the interpretability of the mediation effect;
2. the set of coarse features included in the movie editing should not be considered as “computational modeling”, since they retained low complexity and limited capacity to describe neural systems and cognitive processes;
3. movie editing descriptors represented a coarse set of collinearities across senses, and their relatively large effect size in STS/STG further corroborated the hypothesis that these regions still responded to correspondences across modalities.

Moreover, we would like to further highlight these additional points:

1. this issue is intrinsic to the naturalistic stimulation itself, since such a paradigm captures information that is often shared across distinct sensory modalities (please refer to^46^ for a detailed discussion on the role of formal cinematic features as the editing technique);
2. during the model testing and feature selection procedures in the AV multisensory condition (Supplementary Fig.1), the cleaned models were still able to explain a reasonable portion of variance and the cortical distribution of all sets of features is consistent with previous observations, even for the multimodal high-level representations^47^;
3. beyond early visual areas, the ability to explain brain activity through computational models is far from reaching the noise ceiling^48^.

**Supplementary Methods**

**Behavioral assessment**

The following psychometric scales were administered to participants: one concerning manual dexterity, assessed through the Edinburgh Handedness Inventory^49^ and a set of questionnaires related to sensory imagery: the shortened version of the Bett’s Questionnaire upon Mental Imagery^50^ the Visual Vividness Imagery Questionnaire (VVIQ)^51^ and the Plymouth Sensory Imagery Questionnaire^52^ .

**Computational models**

The following sections will review the models that were adopted in the present work to extract the movie low-level (visual and auditory) and high-level (semantic and categorical) properties. Consistently with the theoretical framework of hierarchical sensory processing^42,53,54^, we exploited the richness of the naturalistic stimulation to investigate stimulus-driven brain responses to low-level, high-level and categorial movie features. Therefore, low-level descriptors for the early visual and auditory systems was adopted to extract the frequential signal properties and their modulation in time of both the visual and auditory movie stimuli: features generated from image GIST and motion energy defined the low-level visual model; sound power spectrum and envelope, the low-level acoustic model. Additionally, two semantic high-level models were employed by combining information from the stimulus semantics (i.e., word2vec algorithm trained in an Italian corpus^55^, and GPT-3^56^ trained on an English corpus) as well as the manual tagging of the categorical content of the visual and auditory movie for event discrimination (i.e., Animals, Houses, Objects, Person, and Vehicles -for the visual stimulus and the auditory track). Finally, a movie editing model was defined on the stimulus properties (i.e., Cuts, Scenes, Dialogues, Audio Descriptions, Soundtracks), introduced during the editing phase which captured coarse slow-paced collinearities between auditory and visual streams, having an impact on both low-level and high-level semantic descriptors.

**Low-level visual model: GIST feature space**

A scene GIST model^57^ was used to quantify the spatial properties of the movie frames convolving a set of Gabor-like filters with a specific frequency and orientation to the image. Each movie frame was segmented into a 4x4 grid and the responses to Gabor filters having four different sizes and four orientations was sampled, resulting in a model comprising 256 features for each frame (as in^58^). Each feature represented the total energy at a particular orientation and spatial frequency, averaged over a position of the visual field. Subsequently, GIST descriptors across 50 frames within 2 seconds were averaged to match the temporal resolution of fMRI. Subsequently, descriptors were normalized, and a Principal Component Analysis (PCA) was applied to retained components able to explain at least 90% of the total variance, thus reducing the model to 22 dimensions. Finally, the remaining columns were convolved with a standard gamma function as the hemodynamic response function.

**Low-level visual model: Motion energy feature space**

The total motion energy was computed for each two seconds of the movie through a set of 4,715 motion energy descriptors consisting of a quadrature-pair of space-time Gabor filters (e.g., Gabor wavelets with three different temporal frequencies at 0 -static energy-, 2, and 4 Hz as in^40^.

MATLAB code is available here: <https://github.com/gallantlab/motion_energy_matlab>.

The model described each movie frame by a set of preferred spatial frequencies, orientations and temporal frequencies that grasp fast-changing visual information. Subsequently, descriptors were normalized and a PCA was applied to retained components able to explain at least 90% of the total variance, thus reducing the model to 398 dimensions. Finally, the remaining columns were convolved with a standard gamma function as the hemodynamic response function.

**Low-level auditory model: Power Spectrum feature space**

Spectral features extraction was carried out following the method described by^42^. We estimated the signal power spectrum for each run through the Welch’s power spectral density estimate^59^ with a Gaussian window (SD of 5 ms, length 30 ms, 1 ms spacing between window) over portions of the signal of 2 seconds length (in order to match the fMRI temporal resolution). The output is a 449-dimensional vector that summarizes the signal power spectrum (expressed in dB units) in the range of 0 Hz to ~15000 Hz computed in bands of 33.5 Hz. For further details about the parameters used please refer to^42,58^. Subsequently, descriptors were normalized and a PCA was applied to retained components able to explain at least 90% of the total variance, thus reducing the model to 5 dimensions. Finally, the remaining columns were convolved with a standard gamma function as the hemodynamic response function.

**Low-level auditory model: Envelope feature space**

To model sound amplitude changes over time, the soundtrack envelope power spectral density was extracted. First, we first evaluated the upper and lower root-mean-square envelopes of the raw sound signal averaged over the two channels, through the MATLAB function *envelope* (option ‘*rms*’) with a sliding window of 10 ms length. The signal power spectrum was estimated over 2 seconds signal bins with the Welch’s power spectral density estimate (Gaussian window, SD 800 ms, length 1 s, 0.5 s spacing between windows). The output is a 49-dimensional vector that summarizes the envelope power spectrum (expressed in dB units) in the range of 1 Hz to 99 Hz computed in bands of 2 Hz. For further details, please refer to^60^. Subsequently, descriptors were normalized and a PCA was applied to retained components able to explain at least 90% of the total variance, thus reducing the model to 8 dimensions. Finally, the remaining columns were convolved with a standard gamma function as the hemodynamic response function.

Therefore, the visual model was represented by a matrix of 1,614 rows (as the number of timepoints of the fMRI) and 420 columns (i.e., features), whereas the acoustic model comprised 1,614 rows and 13 columns.

**High-level model: Semantic feature space using Word2Vec in Italian**

Given the transcript of the whole verbal content of the movie, comprising all the spoken parts (dialogues, monologues, narrator voice and even animal sounds), we manually tokenized it and removed a series of common stop words (e.g., articles, prepositions and pronouns). Note that only nouns, verbs, adjectives, adverbs and onomatopoeic words were spared and then used to derive the semantic representation of the story. We decided to include also onomatopoeic paralinguistic utterances because they directly mimic specific non-speech and non-musical sounds (produced by nature, animals or human activities), whose source is easily recognizable and inherently *signify* what they refer to. The semantic representation of each term in the transcript was derived through word2vec, a word embedding technique^55^ and based on the distributional properties of words in a large corpus of text. Thus, to identify the movie semantic features, we used the itWaC corpus^61^. The itWaC corpus consisted of 2 billion Italian words extracted from the Web (<http://wacky.sslmit.unibo.it/doku.php?id=corpora>), and it was the largest Italian corpus currently available. Therefore, we used the *word2vec* algorithm to obtain the embedding space (128 dimensions, window 5, cbow architecture, pre-trained in^62^). These word-embeddings were the most accurate representation of the Italian language at the beginning of our experiment (2019) and won the Best System Award at the EVALITA 2018 (https://www.evalita.it/evalita-2018/), which is a periodic evaluation campaign of Natural Language Processing tools for the Italian language. Using these word-embeddings, we extracted for each 2 seconds interval (fMRI temporal resolution) a 128-sized vector obtained by averaging all the word vectors included in the time frame. Subsequently, descriptors were normalized and a PCA was applied to retained components able to explain at least 90% of the total variance, thus reducing the model to 72 dimensions. Finally, the remaining columns were convolved with a standard gamma function as the hemodynamic response function.

**High-level model: Compositional semantic features using GPT-3 in English**

Considering the availability of the whole verbal content of the movie in English, the narrative was splitted according to the subtitles, which were mostly based on single complete sentences. Subsequently, we obtained contextual word-embeddings from each sentence using pre-trained GPT-3^56^ with OpenAI API (https://openai.com/) for academic access. GPT-3 is the most advanced artificial language model available nowadays. Its previous version, GPT-2^63^ , was considered the best biologically feasible framework to predict neural activity during language tasks^64,65^. GPT-3 shared the same model and architecture of GPT-2, but consisted of 175 billion parameters, a 100x increase as compared to the previous version.

Briefly, we obtained a vector of 12,288 dimensions from each sentence using the model *text similarity davinci 001* (<https://beta.openai.com/docs/guides/embeddings>).

Sentence-based vectors were stacked in time and resampled to a temporal resolution of 2 seconds, and a PCA was performed to retain at least 90% of the total variance, thus reducing the model to 232 dimensions. Finally, the remaining columns were convolved with a standard gamma function as the hemodynamic response function.

**High-level model: Categorial features space**

Category selective regions in the brain are known to be tuned for processing specific classes of stimuli^2,66-70^. Here, we evaluated the specific contribution of non-linguistic, high-level semantic information in modulating brain activity. We relied on previous literature on the topic^71^ for the definition of the visual categories that, after being validated through comparison with the image segmentation output of an automatic algorithm (see below Visual categorial model validation), were used to label the categories of the auditory track as well.

For the visual condition the continuous stream of information was classified in seven categories: *Animals, Body-parts, Faces, Houses, Objects, Persons, and Vehicles*. The very same tagging procedure was applied to the auditory stimulus as well but instead of using all the seven visual categories, we used the notation based on five major classes derived from the validation procedure. Note that, the two stimulation conditions intrinsically vary in the degree of detail conveyed by the diverse sensory modalities: although the presence of the narrator is meant to describe visual information through speech, the acoustic content still communicates information at a broader scale than the visual one. Think about a dialogue between two characters: in the visual condition is possible to appreciate either the *Face* appearance, the *Person* silhouette, posture, clothing or a specific *Body-part* while in the auditory setting we can just say, globally speaking, that a *Person* is present (a general idea/mental representation of a man or a woman which appearance is up to the listener). These modality-dependent aspects and the need for consistency in the methods across stimulus conditions motivated the choice of the following five auditory categories: *Animals, Houses, Objects, Persons, and Vehicles*. Everyday life hearing depends on the selection of informative sounds among less-relevant background noise. Therefore, our classification was mainly focused on non-stationary foreground sounds, namely those sounds whose signal statistics change over time and result to be more informative of the world around us. However, previous work showed that the presence of background environmental “noise” differentially affects primary and non-primary auditory areas responses to concurrent foreground sounds^72^. Additionally, we can assume that background natural sounds represent a reliable source of information for blind individuals. For these reasons, the classification was also extended to include audio signals from nature, animals, man-made objects and human activities^73^.

**Annotation procedure.** One of the authors of this study carried out the tagging procedure. The stimulus was explored run by run: the relevant features were manually annotated along with their timecode (time resolution of one second).

**Visual categories.** For each time window of 1 second, the annotator wrote down the elements located in foreground and when present, also additional items that for their appearance (color, size, change in position) or story relevance (key information, main characters) capture the viewer's attention. Each entry was then properly classified according to the following seven categories.

*Animals:* all species of animals represented in the movie.

*Body-parts:* it applies only to a given part of the body appearing in isolation and foreground, except from faces*.* A leg, toe or hand are examples of body-parts.

*Faces*: all the faces represented in foreground regardless of viewpoint and lighting. Faces visible from distance and in presence of the trunk or the entire body are not considered falling in this category (see person).

*Houses*: it refers both to a façade of a building in isolation and to groups of edifices or cityscapes. It also comprises other kinds of structures that are not “houses” in a narrow sense but still, pertains to the more general concept of “buildings” (e.g., a farm, a castle).

*Objects*: it collects man-made objects and tools.

*Persons:* it includes the images in which the complete silhouette of the body is visible, or the head and the upper body. Faces in isolation are not falling into this category.

*Vehicles:* this category is meant to include all “means of transportation” represented in the movie (e.g., a car, a bicycle, a truck) or parts of them sufficiently big and detailed to be recognized as pertaining to a specific kind of vehicle (e.g., a bicycle handlebar or tire; a car hood).

Subsequently, visual categorial descriptors were resampled to match the temporal resolution of fMRI and normalized. Then a PCA was applied to retained components able to explain at least 90% of the total variance, thus reducing the model to 5 dimensions. Finally, the remaining columns were convolved with a standard gamma function as the hemodynamic response function.

**Visual categorial model validation.** To validate the quality of the categorial tagging, we ran an automatic labeling of the content of the visual scenery and tested the degree of classification similarity between the two methods. We relied on a specific kind of a pre-trained convolutional neural network (CRF-RNN)^74^ to solve image segmentation and classify the elements of the visual display into five categories (*Animals, Houses, Objects, Person, Vehicles)*. In this approach, pixel-level labels are predicted combining the strengths of Convolutional Neural Networks (CNNs) technique with Conditional Random Fields (CRFs)-based probabilistic graphical modeling. The model comprises two stages: an initial full convolutional deep network followed by a CRF-RNN step, that can be effectively used to accomplish categorial image segmentation tasks. Since the neural net labels slightly differ from the classes that we manually labelled, some categories were grouped together to compare the two approaches. Therefore, aggregated person, faces and body-parts were aggregated in a single descriptor called *whole-person* to adjust for the absence of such finer distinctions in the classes provided within the CRF-RNN (where *person* refers to all the previous). On the other hand, we clustered the net labels referring to the same superordinate category (i.e., airplane, bicycle, boat, bus, car, motorbike, train into *vehicles*; bird, cat, cow, dog, horse, sheep into *animals*; bottle, chair, dining table, potted plant, sofa, tv monitor into *objects*). The rank correlation (Spearman’s ρ) was computed to assess reliability across the two classification methods: *animals* (ρ = 0.518, CI 95 = 0.476 0.559), *objects* (ρ = 0.153, CI 95 = 0.098 0.211), *vehicles* (ρ = 0.384, CI 95 = 0.321 0.442); *persons* (ρ = 0.616, CI 95= 0.580 0.650). Discrepancies in the classification among the two procedures were carefully reviewed by two authors of the present work who double-checked and assessed the goodness of the manual tagging.

**Auditory categories.** The categorial content of the auditory movie was described following the same method that we applied for the visual stimuli. Therefore, the auditory track of each run was sampled at the time resolution of one second and the salient sounds were labeled based on the category they pertain to. Aiming for consistency across models, we adopted the same categories that were used for the labeling of the visual movie after the validation step. Therefore, each sound was classified according to one of the following labels.

*Animals:* all kinds of animal sounds audible in foreground or still clearly recognizable from the background audio track.

*Houses*: it refers to the descriptions of houses, buildings or cityscape appearance. Usually made by the narrator's voice, such portrayals make explicit reference to the presence of a building (not necessarily “houses” strictly speaking). Examples are: “in front of Anita’s house”, “outside, from the castle gate”.

*Objects:* it collects overt reference to objects or refers to sounds that are generated by man-made objects or tools (e.g., the bell ringing, the shower, the teacups chinking).

*Persons:* the presence of a person is mainly denoted by speech or dialogues. Moreover, this category applies to descriptions of a person's appearance and to those sounds that can be unmistakably attributed to a human being (e.g., footsteps, cough, background chattering, screaming).

*Vehicles:* this category includes vehicles' sounds and descriptions of their appearance. All the onomatopoeic sounds recalling vehicles are included in this category (e.g., “wrooooom”, “beep”, “slam”, “screech”).

Subsequently, auditory categorial descriptors were resampled to match the temporal resolution of fMRI and normalized. Then a PCA was applied to retained components able to explain at least 90% of the total variance, thus reducing the model to 4 dimensions. Finally, the remaining columns were convolved with a standard gamma function as the hemodynamic response function.

Therefore, the high-level semantic model based on Word2Vec was represented by a matrix of 1,614 rows (as the number of timepoints of the fMRI) and 81 columns. Moreover, the high-level semantic model based on GPT-3 was represented by a matrix 1,614 by 241. All models (i.e., auditory, visual, Word2Vec semantic, and GPT-3 semantic) were also used in an encoding procedure to predict brain activity of fMRI data from the multisensory AV condition. This step tested the overall quality of the models (Supplementary Fig.1a), as well as it gave us the opportunity to perform a feature selection procedure, further reducing the dimensionality of each of them to a small set of predictors (i.e., 13; please refer to the *Methods* section, *Computational modeling* paragraph, in the main manuscript).

**Movie editing model**

Movies are complex stimuli not only for the multifaceted information they convey but also because of their formal architecture, as it results from the work of the film editor. Indeed, the stylistic choices (e.g., camera’s cuts selection, scenes arrangement and duration) build up the peculiar features of the movie framework that, possibly, influence brain activity (e.g., a change in the scene setting may correspond to modifications in the image luminance and be associated with corresponding adjustments in the music). Moreover, we contributed to the process of editing as well, shortening the original duration and modifying both the auditory and the visual streams. To investigate whether these formal aspects influence movie perception, rather than focusing on the content of the stimulus, we explored the structure of the stimulus in order to model what we called the “movie editing” features. With this term, we thus refer not only to the editor choices already present in the original version, but also to the major modifications that we introduced, namely the inclusion of the audio descriptions and subtitles. We modeled the movie editing features using the same approach devised for the category: four visual (i.e., cuts, scenes, subtitles and text embedded in the visual frames) and three auditory (i.e., audio descriptions, dialogues and soundtracks) movie editing classes were binary tagged in a window lasting 1 second, as reported below.

*Cuts*: this term is used to define sudden changes in camera angle, location and placement from one shot to the following. These events occur frequently during the narration and can be easily spotted in a glance.

*Scenes*: this label refers to the major changes in the story setting (location, characters, actions and time). Thus, we considered a scene as a story unit that takes place in a specific location and in a defined period of time. Compared with cuts, these events happen on a slower timescale.

*Subtitles:* it reflects the story script including all the spoken parts: voice-over, dialogues and environmental sound (mostly animal sounds).

*Text:* all written text readable on the screen *and* belonging to the original movie (it does not include the subtitles that we added afterwards to the visual version of the movie).

*Audio descriptions:* all the parts of the movie script reported by the narrator voice-over. These descriptions are meant to convey the salient aspects of the tale that cannot be inferred merely by listening to the original movie auditory track because they are generally conveyed by the visual scenery. Indeed, audio descriptions contain mostly scenes portrayals for better contextualization and characters actions/emotional state depiction. Note that this category does not include dialogues, environmental sounds and music.

*Dialogues:* it refers only to the part of the discourse pronounced by a person. This includes both conversations, and “monologues” (e.g., the reporter announcing the news to the spectators, the priest speaking to the audience in the church).

*Soundtracks:* background music tracks.

Subsequently, movie editing descriptors were resampled to match the temporal resolution of fMRI and normalized. Then a PCA was applied to the retained components able to explain at least 90% of the total variance, thus reducing the model to four dimensions. Finally, the remaining columns were convolved with a standard gamma function as the hemodynamic response function.

The goodness of this model was tested in an encoding procedure to predict brain activity of fMRI data from the multisensory AV condition (Supplementary Fig.2a). Moreover, we measured the collinearity between this model and all the others Supplementary Fig.2b).

Since movie editing features share a large portion of variance across all the other models, we decided to orthogonalize all the features for the stimulus properties related to the film editing process. Finally, we re-tested the overall quality of these “cleaned” models in an encoding procedure to predict brain activity of fMRI data of the multisensory AV condition (Supplementary Fig.1b). Similar to the original models, this procedure further reduced the dimensionality of each of them to a small set of predictors (i.e., 13; please refer to the *Methods* section, *Computational modeling* paragraph, in the main manuscript).

**Correspondences across visual and auditory features**

The relationships in the time frequency space between the visual and auditory streams of the stimulus were examined at the maximum available temporal resolution (i.e., frame duration, which was 0.04 s at 25 frames per second). Here, to measure finer co-occurrences in time between the two streams, we relied on fast static visual features extracted from each frame using the GIST model^57^, and the soundscape acoustic spectral properties^42^. Subsequently, since both models retained high dimensionality which prevented the calculation of an overall measure of coherence between them, two representational similarity matrices (RDM) were built by measuring the Euclidean distances of each timepoint in the acoustic and visual feature spaces. As results, these two RDM described the relationship between each movie frame in the visual and auditory domains. To further reduce the dimensionality of the RDMs, the weighted contribution of each frame was evaluated by summing the Euclidean distances with all the other frames. This procedure, which is analogous to the weighted degree of a node in a fully connected graph, generated a time series for each model which resembled the similarity of movie frames across time. Finally, the time series generated from the visual features were compared with the one obtained from the acoustic ones by means of a Continuous Wavelet Transform^75^ (CWT, default parameters with the Morlet wavelet). Results were depicted in Fig.1c and indicated thousands of timepoints showing high coherence, suggesting the visual and acoustic features were continuously entangled representing common events lasting from tenths of a second to several minutes.

**Encoding in the AV condition**

Since model dimensionality was largely different across computational models (from a few columns of the acoustic model to hundreds in the visual one), an encoding procedure was performed in the multisensory AV condition only, to both verify the goodness of our descriptors and to prune irrelevant features.

Specifically, we first defined an outer cross-validation leave-one-run-out loop in which a run was used as a test set, whereas the other five as training set. In the training set, we defined another inner cross-validation leave-one-run-out loop in which a run was used as a validation set to tune the model parameters (i.e., the smallest set of features which provided the best prediction). During the training phase, for each step of the inner loop, a voxel-wise multiple regression was performed to obtain t-stats of the beta coefficients of model descriptors. The t-stats were calculated by averaging the beta coefficients across subjects and by dividing them for their standard errors. The inner cross-validation procedure generated an averaged across folds t-stat for each feature. Within the inner loop, feature selection was performed by considering the prediction performance (R^2^) of reduced models, obtained by increasingly adding the best features (i.e., the ones with the highest t-stats) from one to six. We decided to limit the maximum number of features which could predict a BOLD signal in a voxel to six, as the encoding properties of small patches of cortex (or voxels) seems to rely on a relatively low dimensionality^76,77^. Then, the selected features were used to predict the test set of the outer cross-validation loop, thus, to provide an unbiased estimation of the goodness of fit. Unthresholded R^2^ maps were reported in Supplementary Fig.1 and demonstrated the overall quality of our encoding procedure across all the models.

Finally, for each model, we retained only the best and most frequently used thirteen features across brain voxel to match the dimensionality of our smallest set of descriptors (the acoustic model). The selected acoustic, visual and semantic features, with equal dimensionality and high predictive power, were used on independent data (i.e., model-mediated ISC in the A *vs* V condition, both in TD and SD participants).

In addition, an overall measure of collinearity (R^2^) was estimated between our final set of computational features, before and after the removal of the movie editing features, thus accounting for the residual collinearities between the models. In detail, each pair of models were compared so that each model acted as a predictor of the other and vice versa. To do so, the multiple regression was combined with a bootstrapping procedure (10,000 iterations) to randomly sample four columns from the predictor model (i.e., four is the size of the movie editing model which retained the lowest dimensionality) and one column from the predicted model. Ultimately, this procedure generated a predicted model which was compared to the original one by means of R^2^ to obtain a final unbiased estimation of collinearity between models of different dimensionality. Note, that after having cleaned the movie editing features from all the other models, as expected, they still retained some degree of collinearity (up to 5% of total variance between visual and acoustic streams) one with the others (Supplementary Fig.2).

**Supplementary Figures**
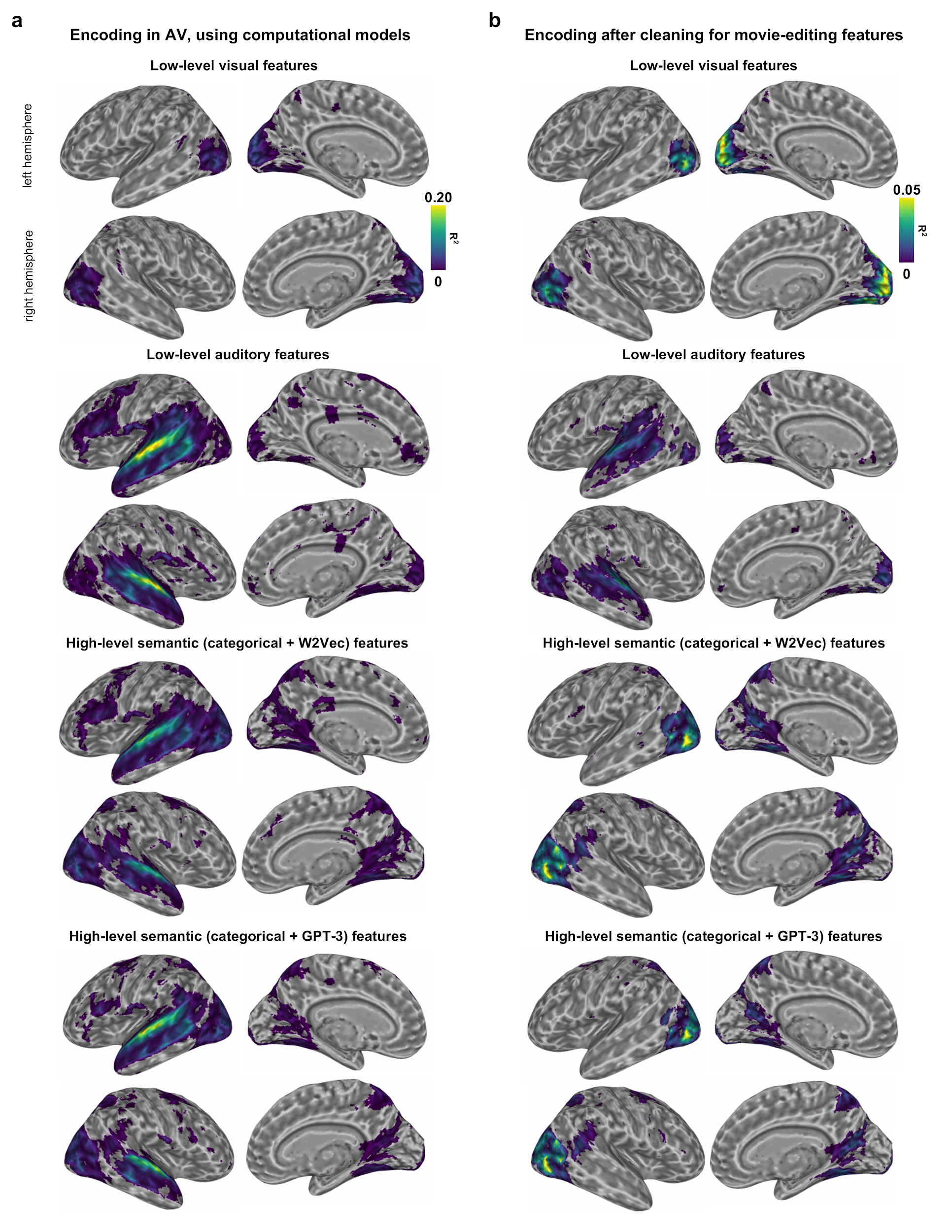


**Supplementary Fig.1: Encoding of computational models in the AV condition.**  Figure depicts the results of the encoding procedure on the AV condition, for the low-level visual, low-level acoustic, high-level Word2Vec semantic (based on categorical information and Word2Vec embeddings of Italian words) and the high-level GPT-3 semantic (based on categorical information and GPT-3 embeddings of English sentences) models, respectively. Panel **a** represents the explained variance for the original models, whereas **b** shows the performance of models when a large portion of common variance is removed (i.e., movie editing features). Unthresholded cross-validated R^2^ maps (cross-validated R^2^ > 0, minimum cluster size of 20 adjacent voxels), averaged across subjects, indicate an overall high quality of the encoding procedure for different computational models and for both original and cleaned computational features. Peak of cross-validated R^2^ (averaged across subjects) for low-level visual features: right Superior Occipital Gyrus, R^2^ = 0.127 at MNI_xyz_: 19 -89 25 for the original model, right Superior Occipital Gyrus, R^2^ = 0.117 at MNI_xyz_: 19 -89 25 for the cleaned model. Peak of R^2^ for low-level auditory features: left STG, R^2^ = 0.290 at MNI_xyz_: -65 -11 1 for the original model, right STG, R^2^ = 0.043 at MNI_xyz_: 58 -14 4 for the cleaned model. Peak of R^2^ for high-level semantic (categorical and Word2Vec) features: left STG, R^2^ = 0.195 at MNI_xyz_: -65 -11 1 for the original model, right Middle Occipital Gyrus, R^2^ = 0.076 at MNI_xyz_: 52 -77 1 for the cleaned model. Peak of R^2^ for high-level semantic (categorical and GPT-3) features: left STG, R^2^ = 0.231 at MNI_xyz_: -65 -11 1 for the original model, right Middle Occipital Gyrus, R^2^ = 0.080 at MNI_xyz_: 52 -74 4 for the cleaned model.


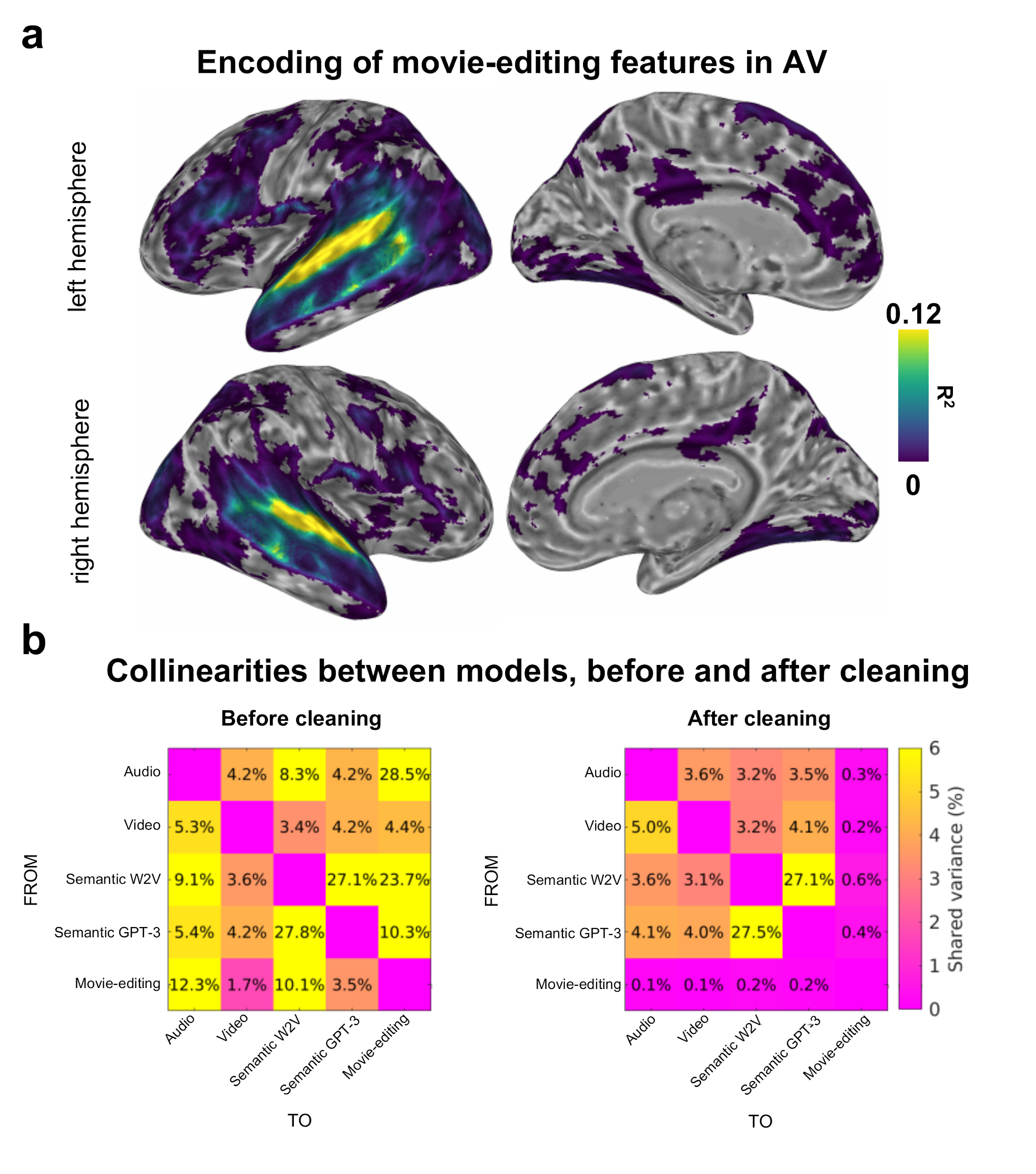


**Supplementary Fig.2: The role of movie-editing features.** Panel **a** shows the unthresholded cross-validated R^2^ map (cross-validated R^2^ > 0, minimum cluster size of 20 adjacent voxels), averaged across-subjects, for the encoding of the movie editing features during multisensory AV stimulation. Peak of R^2^ for movie editing features: left STG, R^2^ = 0.285 at MNI_xyz_: -65 -11 1. The matrix on the left in **b** demonstrates that our set of stimulus features are collinear and therefore share a large percentage of variance. Since both low-level and high-level models are affected by the coarse properties related to the movie editing descriptor, each of the above was orthogonalized by the latter with the aim to clean them from the portion of common variance. Residual collinearities across models after cleaning are shown in the matrix on the right side of **b**. Model collinearities remain high for the two alternative high-level semantic descriptors, one based on Italian word-embeddings obtained through Word2Vec (W2V), and one obtained from English embeddings using GPT-3.


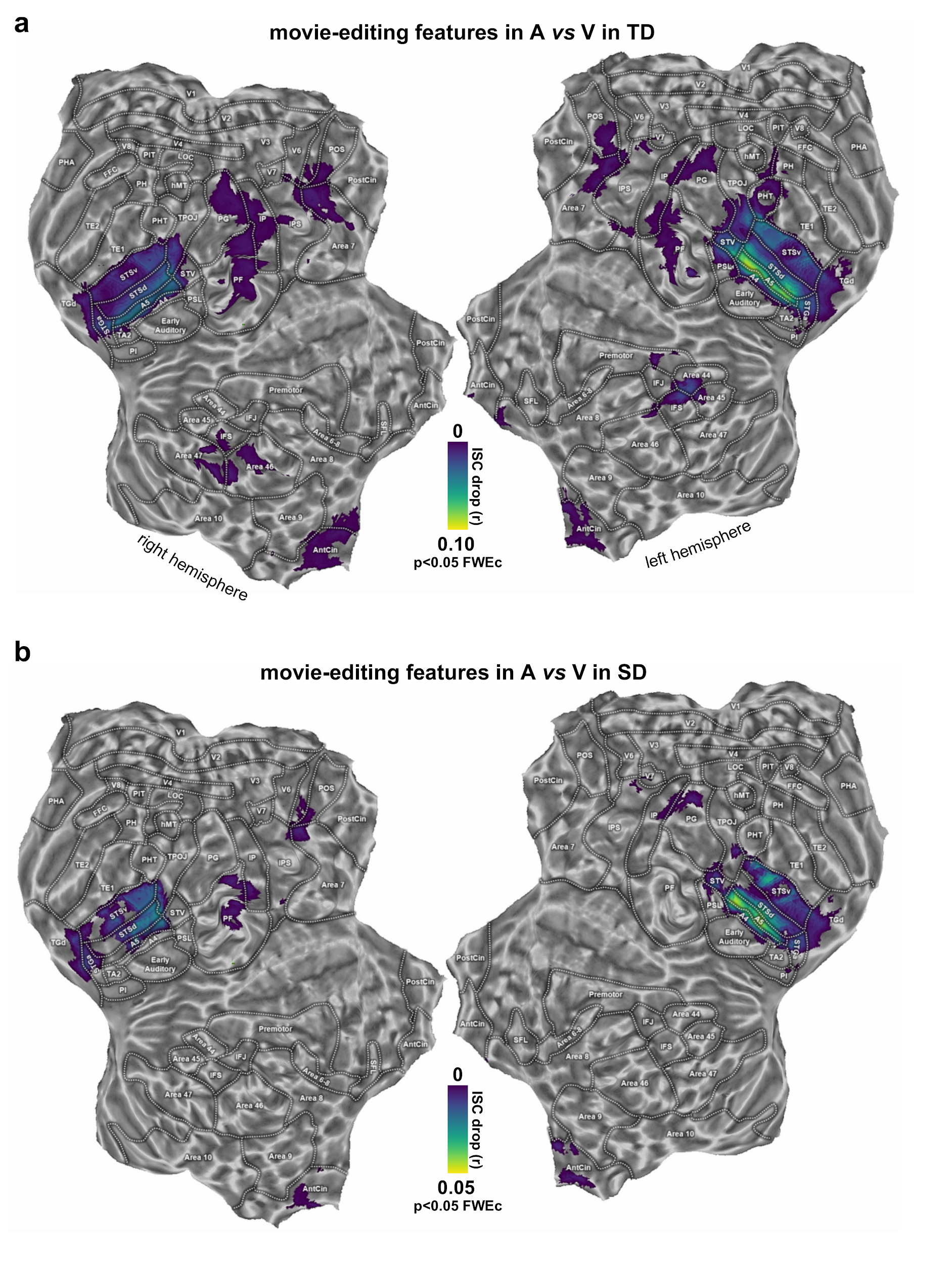


**Supplementary Fig.3: The role of movie-editing features in the model-mediated ISC in the A *vs* V conditions.** In **a**, we report the model-mediated ISC across TD in the A *vs* V condition for the set of movie editing features (p < 0.05, one-tailed, Family-wise Error corrected -FWEc-, minimum cluster size of 20 adjacent voxels). In **b**, the same mediation effect is represented across SD participants. All results were corrected for multiple comparisons (p < 0.05, one-tailed, FWEc, minimum cluster size of 20 adjacent voxels). Results indicated that the movie editing coarse description (e.g., film cuts, scene transitions, presence of dialogues, and music), which shared a large portion of variance with both the low-level and the high-level features (see Supplementary Fig.2), was an effective moderating variable: indeed, it was able to account for roughly half the magnitude of ISC, and its effect was consistently larger as compared to the other computational models (Fig.4). Peak of ISC drop: TD A vs V in left STS: r = 0.098, 95th range = 0.019 0.248, MNI_xyz_: -68 -35 4; deprived A vs V in the left STS: r = 0.057, 95th range = 0.001 0.211, MNI_xyz_: -62 -32 1.


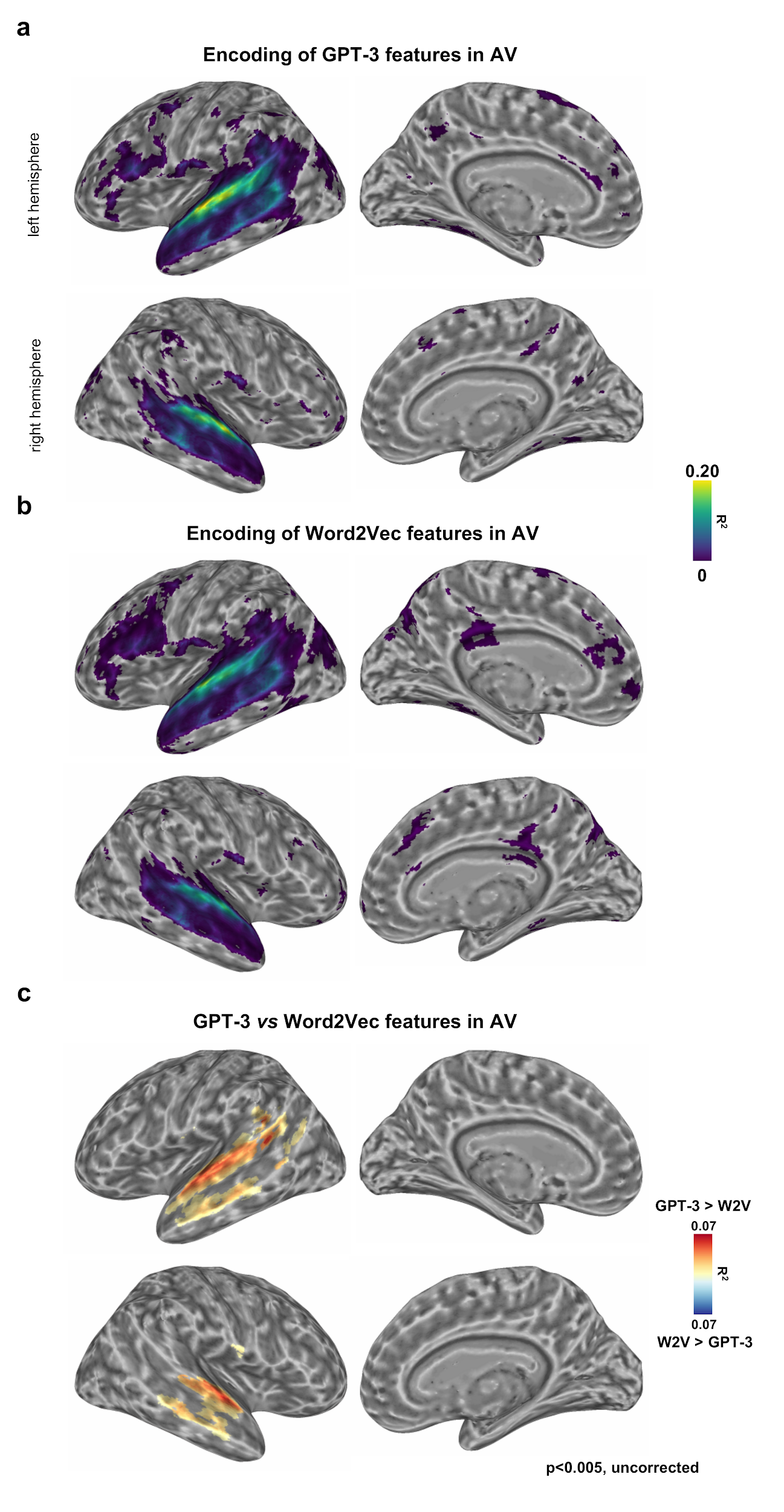


**Supplementary Fig.4: Language models.** Here we report the results of our encoding procedure by using only the features from GPT-3 and Word2Vec algorithms. The aim of this control analysis was to test whether artificial language models based on compositionality retained an increased explained variance as compared to traditional word-embeddings^64^. Panels **a** and **b** show unthresholded cross-validated R^2^ maps (cross-validated R^2^ > 0, minimum cluster size of 20 adjacent voxels), averaged across-subjects, for the encoding of GPT-3 and Word2Vec language related features, respectively. Peak of R^2^ for GPT-3 features: left STG, R^2^ = 0.236 at MNI_xyz_: -65 -14 1. Peak of R^2^ for Word2Vec features: left STG, R^2^ = 0.194 at MNI_xyz_: -65 -11 1.

Panel **c** reports the difference between R^2^ maps of these two models (Wilcoxon signed rank test, p < 0.005, two-tailed, uncorrected for multiple comparisons, minimum cluster size of 20 adjacent voxels). The results highlight the ability of GPT-3 to explain large portions of variance (up to 7.6% of BOLD activity in posterior STS at MNI_xyz_: -56 -44 11) in the audio-visual condition in language related regions (bilateral STG and STS). Two voxels in left posterior STG (0.3% of the total volume) showed a significantly higher explained activity for Word2Vec as compared to GPT-3.


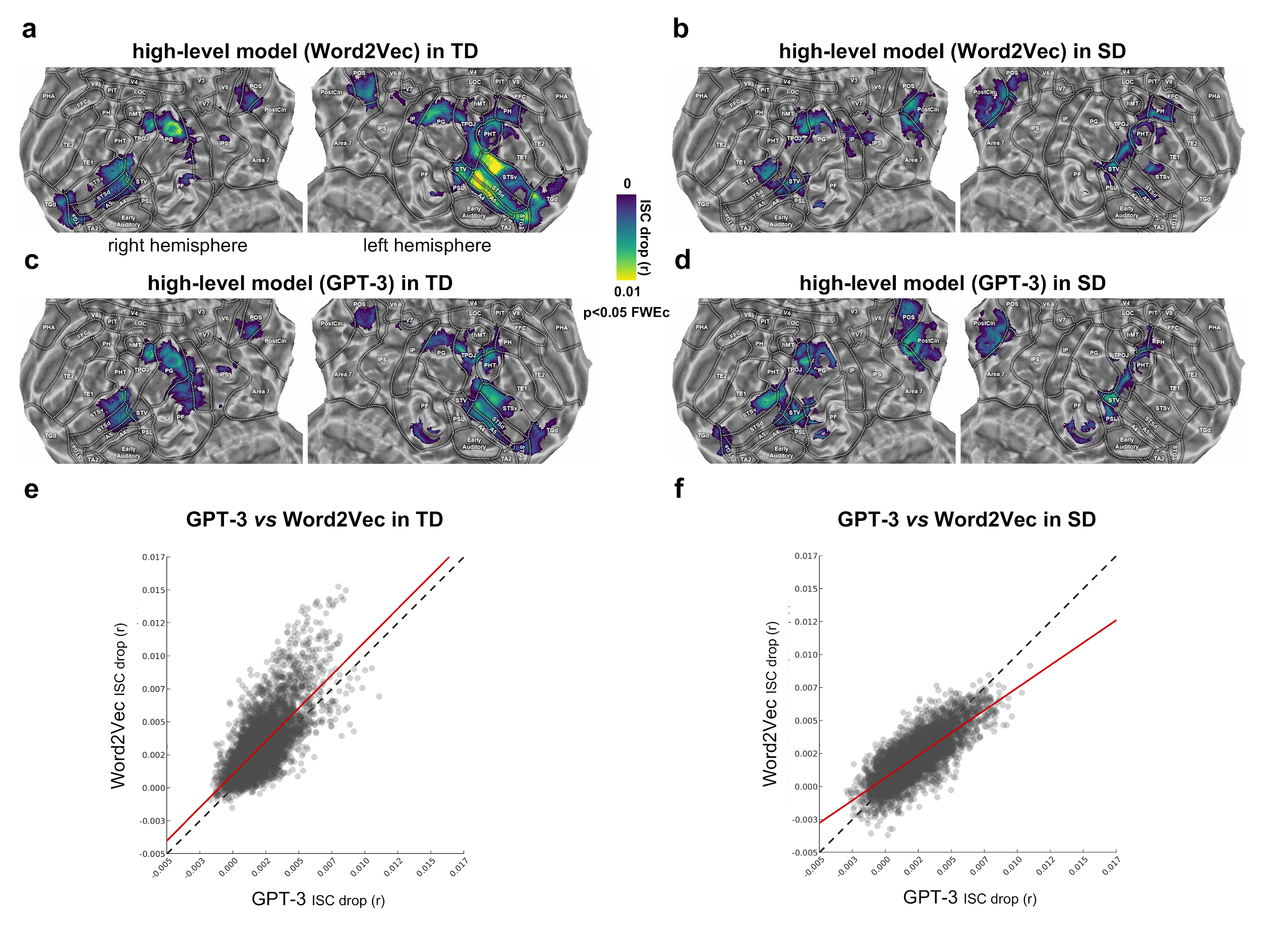


**Supplementary Fig.5: Comparison between high-level models based on Word2Vec and GPT-3.** Panels **a** and **b** report the model-mediated ISC across the A *vs* V condition in TD and SD participants for the high-level model based on Word2Vec semantic features (i.e., categorical information and Word2Vec features; p < 0.05, one-tailed, Family-wise Error corrected -FWEc-, minimum cluster size of 20 adjacent voxels). Panels **c** and **d** show the same condition for the high-level model based on GPT-3 semantic features (i.e., categorical information and GPT-3 features, p < 0.05, one-tailed, FWEc, minimum cluster size of 20 adjacent voxels). Panels **e** and **f** report the scatter dot plots and regression lines (in red) to compare ISC drops during mediation between GPT-3 and Word2Vec in TD and SD participants. Each dot represents a voxel in the conjunction mask represented in Fig.2c. Although GPT-3 model shows higher ability to predict fMRI activity in the AV multisensory condition (see Supplementary Fig.4), when used as a mediator in the ISC between the audio-only and video-only conditions, it retains similar effect sizes and spatial mapping as compared to Word2Vec.


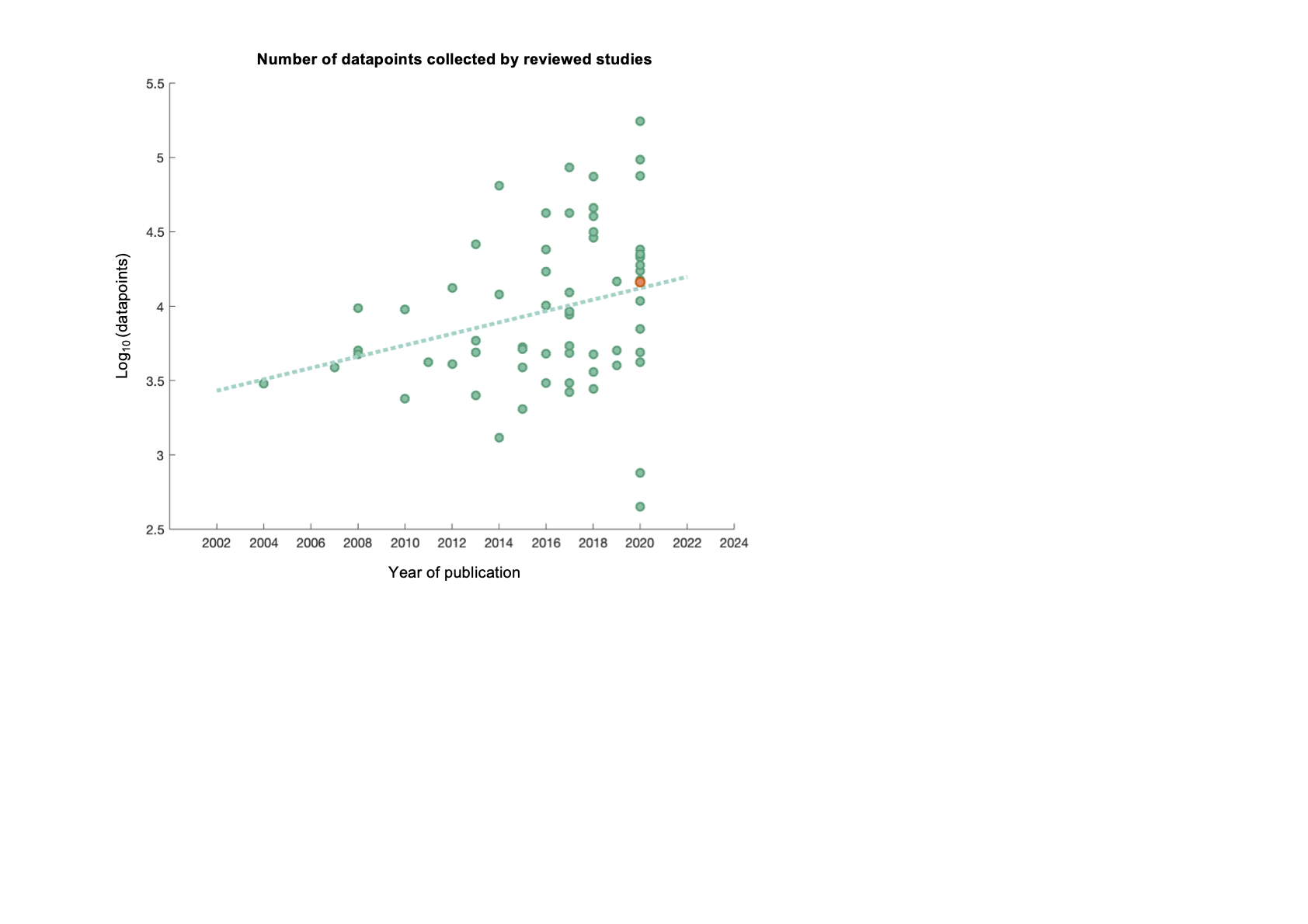


**Supplementary Fig.6: ISC sample sizes in the fMRI literature.** Number of acquired data points as a function of the year of publication across fMRI studies using ISC. In the scatter plot, each green point refers to a reviewed paper while the orange dot identifies the study presented here. Data are plotted as the number of functional scans multiplied by the number of subjects on a logarithmic (base 10) scale. The number of data points collected in our study align with that of other works relative to year of publication.

Studies were retrieved after querying on PubMed and Google Scholar in September 2020 the keywords: “fMRI”, “ISC”, “naturalistic stimulation”, “movie”, “narratives” and “stories”. Each time a paper matched our search criteria we processed it and we collected the following data (see Table 3): i) authors and year of publication; ii) name of the movie/track (when available); iii) experimental conditions (i.e., auditory, visual, audiovisual); iv) stimulus length (in seconds); v) sample size; vi) fMRI parameters: TR and number of volumes acquired (when specified). This information allowed us to calculate the amount of data points collected in each study, referring only to the sample of interest. When the same dataset was adopted in multiple publications, we decided to report the year of the work published first. In the case of studies performing ISC analysis on previously acquired datasets, we scrutinized the original study and considered it only in the case it already used the ISC method. Otherwise, we just referred to the more recent work adopting ISC analysis (for an example of that see the ref. Nastase et al., 2020 in Supplementary Table 3). The calculation of the total data points was performed dividing the stimulus length in seconds for the TR (or, when available we just considered the number of acquired functional scans) and multiplying the results by the sample size. Note that, since our aim was to recruit a sufficient number of sensory deprived subjects to guarantee reliability of ISC results, the total number of data points of the present work was calculated considering the sample size of blind/deaf participants (N = 9; therefore, the orange dot indicates log_10_ of 1614 functional scans by 9 subjects), although the ISC was performed across the two groups (N = 18).

**Supplementary Tables**

| **Cortical Area Label** | **ROI ID (Glasser et al., 2016)** |
| --- | --- |
| V1 | 1 |
| V2 | 4 |
| V3 | 5,13, 19, 158 |
| V4 | 6 |
| V8 | 7 |
| V7 | 16 |
| V6 | 3, 152 |
| PIT (Posterior InferoTemporal) | 22 |
| FFC (Fusiform Face Complex) | 18 |
| PH (Temporal Cortex) | 138 |
| PHT (Middle Temporal Gyrus) | 137 |
| PHA (ParaHippocampal Cortex) | 126, 127, 155 |
| hMT (human MT) | 2, 23 |
| Early auditory | 24,104, 124, 173, 174 |
| A4 | 175 |
| A5 | 125 |
| STV (Superior Temporal Visual Cortex) | 28 |
| PSL (PeriSylvian Language) | 25 |
| PI (ParaInsular) | 178 |
| STGa (Superior Temporal Cortex - anterior) | 123 |
| STGd (Superior Temporal Cortex - dorsal) | 128, 129 |
| STSv (Superior Temporal Cortex - ventral) | 130, 176 |
| TGd (Lateral Temporal Cortex - dorsal) | 131 |
| TA2 (Auditory Association) | 107 |
| TPOJ (Temporo-Parieto-Occipital Junction) | 139, 140, 141 |
| TE1 (Middle Temporal Gyrus) | 132, 133, 177 |
| TE2 (Inferior Temporal Sulcus/Gyrus) | 134, 136 |
| Area 7 (Parietal Cortex) | 29, 42, 45, 46, 47 |
| POS (ParietoOccipital Sulcus) | 15, 31 |
| PostCin (Post Cingulate Cortex) | 27, 30, 32, 33, 34, 38, 35, 161, 162, 14 |
| PF (rostral Inferior Parietal Cortex) | 147, 148 149 |
| IPS (IntraParietal Sulcus) | 17,48,49,50, 95,117 |
| IP (IntraParietal Cortex) | 144, 145, 146 |
| PG (intermediate Inferior Parietal Cortex) | 143, 150, 151 |
| IFS (Inferior Frontal Sulcus) | 81, 82 |
| IFJ (Inferior Frontal Cortex) | 79, 80 |
| BA44 (Inferior Frontal, pars Opercularis) | 74 |
| BA45 (Inferior Frontal, pars Triangularis) | 75 |
| Area 47 (Inferior Frontal, pars Orbitalis) | 66, 76, 77, 94, 171 |
| Area 46 (Dorsolateral Prefrontal Cortex) | 83, 84, 85, 86 |
| Area 8 (Medial Prefrontal Cortex) | 63, 67, 68, 70, 73 |
| Area 9 (Medial Prefrontal Cortex) | 69, 71, 87 |
| Area 6-8 (Dorsolateral Prefrontal Cortex) | 97, 98 |
| AntCin (Anterior Cingulate Cortex) | 57, 58, 59, 60, 61, 62, 64, 165, 179, 180 |
| Area 10 (OrbitoFrontal Cortex) | 65, 72, 88, 89, 90, 170 |
| PreMot (PreMotor Cortex) | 12, 54, 96, 10, 11, 56, 78 |

**Supplementary Table 1.** Correspondences between cortical labels and the brain parcellation provided in the HCP Atlas (Glasser et al., 2016).

| **Subject** | **Gender** | **Age** | **Cause of blindness** | **Residual Light Perception** | **Age of Braille reading** |
| --- | --- | --- | --- | --- | --- |
| 041 | M | 49 | retinopathy of prematurity | NLP | 6 |
| 039 | M | 42 | retinitis pigmentosa | NLP | 6 |
| 036 | M | 48 | optic nerve atrophy | NLP | 6 |
| 035 | F | 37 | Leber congenital amaurosis | NLP | 6 |
| 038 | M | 32 | retinal detachment | NLP | 6 |
| 043 | F | 41 | bilateral retinoblastoma | NLP | 6 |
| 042 | M | 19 | retinal detachment | NLP | 6 |
| 053 | M | 57 | retinopathy of prematurity | NLP | 6 |
| 033 | F | 68 | optic nerve atrophy | NLP | 6 |

| **Subject** | **Gender** | **Age** | **Cause of deafness** | **First Language** | **Hearing aid use** |
| --- | --- | --- | --- | --- | --- |
| 044 | M | 24 | hereditary | sign | used during childhood |
| 045 | F | 21 | hereditary | sign | used during childhood |
| 046 | M | 24 | hereditary | sign | used during childhood |
| 047 | M | 26 | hereditary | sign | used during childhood |
| 048 | M | 22 | hereditary | sign | used during childhood |
| 049 | F | 18 | hereditary | sign | used during childhood |
| 050 | F | 28 | Sensorineural  hearing loss | sign | currently used |
| 051 | F | 32 | hereditary | sign | used during childhood |
| 052 | F | 22 | hereditary | sign | used during childhood |

**Supplementary Table 2.** Characteristics of congenitally blind and congenitally deaf participants. NLP, No Light Perception; M, male; F, female.

| **Authors** | **Year** | **Stimulus** | **Condition** | **N subs** | **Length (min)** | **TR** | **Datapoints** |
| --- | --- | --- | --- | --- | --- | --- | --- |
| Hasson U., et al. | 2004 | *The Good, the Bad and the Ugly* | audio-visual | 5 | 0:30:00 | 3000 | 600 |
| Golland Y., et al. | 2007 | *The Good, the Bad and the Ugly* | audio-visual | 12 | 0:16:09 | 3000 | 323 |
| Hasson U., et al. | 2008 | *Curb your enthusiasm* | audio-visual | 12 | 0:27:00 | 2000 | 810 |
| Wilson S. M., et al. | 2008 | *Carrotblanca, Hare Do* | only audio | 12 | 0:14:02 | 2000 | 421 |
| Wilson S. M., et al. | 2008 | *Dripalong Daffy, The scarlet Pumpernickel, Box Office Bunny* | audio-visual | 12 | 0:13:14 | 2000 | 397 |
| Kauppi J.P., et al. | 2010 | *Crash* | audio-visual | 15 | 0:36:00 | 3400 | 635 |
| Hasson U., et al. | 2010 | *The Good, the Bad and the Ugly* | audio-visual | 12 | 0:10:00 | 3000 | 200 |
| Lerner Y., et al. | 2011 | *Pieman* | only audio | 15 | 0:07:00 | 1500 | 280 |
| Nummenmaa L., et al. | 2012 | *When Harry Met Sally and The Godfather* | audio-visual | 16 | 0:24:00 | 1737 | 829 |
| Honey C. J., et al. | 2012 | *narrative story* | only audio | 9 | 0:11:21 | 1500 | 454 |
| Salmi J., et al. | 2013 | *The Match Factory Girl* | audio-visual | 13 | 1:07:00 | 2000 | 2010 |
| Boldt R., et al. | 2013 | *Postia Pappi Jaakobille (audio-drama)* | only audio | 13 | 0:18:51 | 2500 | 452 |
| Abrams D. A., et al. | 2013 | *music pieces* | only audio | 17 | 0:09:35 | 2000 | 288 |
| Regev M., et al. | 2013 | *Pieman* | only audio | 9 | 0:07:00 | 1500 | 280 |
| Hanke M., et al. | 2014 | *Forrest Gump* | only audio | 18 | 2:00:00 | 2000 | 3600 |
| Lahnakoski J. M., et al. | 2014 | *Desperate Housewives* | audio-visual | 20 | 0:20:00 | 2000 | 600 |
| Bernardi G., et al. | 2014 | *On-board camera F1 clips* | audio-visual | 10 | 0:05:25 | 2500 | 130 |
| Vanderwal T., et al. | 2015 | *Inscapes, Ocean's Eleven* | audio-visual | 22 | 0:07:20 | 2500 | 176 |
| Kauttonen J., et al. | 2015 | *At land* | only video | 12 | 0:14:40 | 2000 | 440 |
| Herbec A., et al. | 2015 | *Sleeping Beauty ballet* | audio-visual | 16 | 0:10:40 | 2000 | 320 |
| Schmälzle R., et al. | 2015 | *real-life political speeches* | only-audio | 12 | 0:07:05 | 2500 | 170 |
| Simony E., et al. | 2016 | *Pieman* | only audio | 36 | 0:07:00 | 1500 | 280 |
| Simony E., et al. | 2016 | *Twilight Zone* | audio-visual | 24 | 0:25:00 | 1500 | 1000 |
| Chen J., et al. | 2016 | *Sherlock* | audio-visual | 22 | 0:48:00 | 1500 | 1920 |
| Jääskeläinen I. P., et al. | 2016 | *The Circus, City Lights* | only audio | 18 | 0:31:32 | 2000 | 946 |
| Lu K. H., et al. | 2016 | *The Good, the Bad and the Ugly* | audio-visual | 9 | 0:11:14 | 2000 | 337 |
| Chen J., et al. | 2016 | *The Lateness of the Hour from Twilight Zone* | audio-visual | 12 | 0:10:00 | 1500 | 400 |
| Ren Y., et al. | 2017 | *The butterfly circus* | audio-visual | 17 | 0:20:00 | 2200 | 545 |
| Chen J., et al. | 2017 | *Sherlock* | audio-visual | 22 | 0:48:00 | 1500 | 1920 |
| Yeshurun Y., et al. | 2017 | *Pretty Mouth and Green my Eyes* | audio-visual | 19 | 0:11:32 | 1500 | 461 |
| Lahnakoski J. M., et al. | 2017 | *Star Wars, Indiana Jones: Raiders of the Lost Ark; James Bond-Golden eye* | audio-visual | 18 | 0:20:42 | 1800 | 690 |
| Nguyen V. T., et al. | 2017 | *The butterfly circus* | audio-visual | 17 | 0:20:00 | 2200 | 545 |
| Bacha-Trams M., et al. | 2017 | *My Sister's Keeper* | audio-visual | 30 | 1:34:56 | 2000 | 2848 |
| Iidaka T., et al. | 2017 | *Mr. Bean* | only video | 15 | 0:08:25 | 2500 | 202 |
| Yeshurun Y., et al. | 2017 | *Story1, Story2* | only audio | 18 | 0:06:44 | 1500 | 269 |
| Schmälzle R., et al. | 2017 | *movie clips* | audio-visual | 24 | 0:04:35 | 2500 | 110 |
| Jang C., et al. | 2017 | *4 video clips* | only video | 15 | 0:12:04 | 2000 | 362 |
| Thomas R.M., et al. | 2018 | *actions routines movie* | only video | 22 | 0:22:00 | 721 | 1831 |
| Haufe S., et al. | 2018 | *Dog Day Afternoon* | audio-visual | 11 | 0:10:50 | 1500 | 433 |
| Pollick F.E., et al. | 2018 | *13 &32* | audio-visual | 18 | 0:05:08 | 2000 | 154 |
| Parkinson C., et al. | 2018 | *14 clips* | audio-visual | 42 | 0:36:25 | 2000 | 1093 |
| Lankinen K., et al. | 2018 | *At land* | only video | 8 | 0:15:00 | 2000 | 450 |
| Finn E.S., et al. | 2018 | *narrative story* | only audio | 22 | 0:21:50 | 1000 | 1310 |
| Guntupalli J.S., et al. | 2018 | *Raiders of the Lost Ark* | audio-visual | 11 | 2:00:00 | 2500 | 2880 |
| Bacha-Trams M., et al. | 2018 | *My Sister's Keeper* | audio-visual | 26 | 1:34:56 | 2000 | 2848 |
| Nguyen M., et al. | 2019 | *narrative story* | only audio | 18 | 0:07:00 | 1500 | 280 |
| Loiotile R., et al. | 2019 | *Pieman, Taken, Blow Out, The Conjuring* | only audio | 18 | 0:27:02 | 2000 | 811 |
| Blank I. A., et al. | 2019 | *Pieman* | only audio | 19 | 0:07:00 | 2000 | 210 |
| Di X., et al. | 2020 | *Partly Cloudy* | audio-visual | 29 | 0:05:36 | 2000 | 168 |
| Blank I.A., et al. | 2020 | *Pieman* | only audio | 20 | 0:07:00 | 2000 | 210 |
| Visconti di Oleggio Catello M., et al. | 2020 | *The Grand Budapest Hotel* | audio-visual | 25 | 0:50:00 | 1000 | 3000 |
| Fasano M.C., et al. | 2020 | *Piano Sonata K. 98 by Domenico Scarlatti* | audio-visual | 10 | 0:02:31 | 2000 | 76 |
| Sachs M. E., et al. | 2020 | *Discovery of the Camp; Frysta; Race against the sunset (music)* | only audio | 36 | 0:11:08 | 1000 | 668 |
| Salmi J., et al. | 2020 | *Three Wise Men* | audio-visual | 51 | 0:13:37 | 1900 | 430 |
| Hudson M., et al. | 2020 | *The Conjuring 2; Insidious* | audio-visual | 37 | 3:24:58 | 2600 | 4730 |
| Chang C. H., et al. | 2020 | *Story (part C)* | only audio | 25 | 0:17:15 | 1500 | 690 |
| Nastase S. A., et al. | 2020 | *Slumlord, Reach for the Stars One Small Step at a time* | audio-visual | 16 | 0:29:26 | 1500 | 1177 |
| Nastase S. A., et al. | 2020 | *It is not the fall that gets you* | audio-visual | 18 | 0:09:45 | 1500 | 390 |
| Nastase S. A., et al. | 2020 | *Pie man (PNI)* | only-audio | 39 | 0:06:57 | 1500 | 278 |
| Nastase S. A., et al. | 2020 | *Running from the Bronx (PNI)* | only-audio | 40 | 0:09:21 | 1500 | 374 |
| Nastase S. A., et al. | 2020 | *I knew you were black* | only-audio | 40 | 0:13:21 | 1500 | 534 |
| Nastase S. A., et al. | 2020 | *The man who forgot Ray Bradbury* | only-audio | 40 | 0:13:57 | 1500 | 558 |
| Schmälzle R., et al. | 2020 | *Bang! You are dead* | audio-visual | 494 | 0:08:02 | 2470 | 195 |
| Skaribas E., et al. | 2020 | *Giselle’s solo dance in Act II of Giselle* | only video | 10 | 0:01:30 | 2000 | 45 |

**Supplementary Table 3.** Reviewed datasets summarized by: author and year of publication, stimulus used, condition modality, sample size, stimulus duration and number of datapoints acquired (given the fMRI TR).

**REFERENCES**

1. Chen, J. et al. Shared memories reveal shared structure in neural activity across individuals. *Nat. Neurosci.* **20**, 115-125 (2017).

2. Gould Van Praag, C. D. et al. (2017). Mind-wandering and alterations to default mode network connectivity when listening to naturalistic versus artificial sounds. *Sci. Rep.* **7,** 1-12 (2017).

3. Regev, M. et al. Selective and invariant neural responses to spoken and written narratives. *J. Neurosci* **33**, 15978-15988 (2013).

4. Dikker, S. et al. On the same wavelength: predictable language enhances speaker–listener brain-to-brain synchrony in posterior superior temporal gyrus. *J. Neurosci.* **34**, 6267-6272 (2014).

5. Ferstl, E. C. et al. The extended language network: A meta-analysis of neuroimaging studies on text comprehension. *Hum. Brain Mapp.* **29,** 581-593 (2008).

6. Honey, C. J. et al. Slow Cortical Dynamics and the Accumulation of Information over Long Timescales. *Neuron* **76**, 423-434 (2012).

7. Loiotile, R. E. et al. Naturalistic audio-movies and narrative synchronize “visual” cortices across congenitally blind but not sighted individuals. *J. Neurosci.* **39**, 8940-8948 (2019).

8. Ames, D. L. et al. Contextual alignment of cognitive and neural dynamics. *J. Cogn. Neurosci.* **27**, 655-664 (2015).

9. Simony, E. et al. Dynamic reconfiguration of the default mode network during narrative comprehension. *Nat. Comm.* **7**, 1-13 (2016).

10. Noppeney, U. et al. Effects of visual deprivation on the organization of the semantic system. *Brain* **126**, 1620-1627 (2003).

11. Amedi, A. et al. Functional imaging of human crossmodal identification and object recognition. *Exp. Brain Res.* **166**, 559-571, (2005).

12. Koelsch, S. et al. Music, language and meaning: Brain signatures of semantic processing. *Nat. Neurosci.* **7**, 302-307 (2004).

13. Baldassano, C. et al. Discovering Event Structure in Continuous Narrative Perception and Memory. *Neuron* **95**, 709-721 (2017).

14. Santi, A., & Grodzinsky, Y. Working memory and syntax interact in Broca’s area. *NeuroImage* **37**, 8-17 (2007).

15. Porada, D. K. et al. Trimodal processing of complex stimuli in inferior parietal cortex is modality-independent. *Cortex* **139**, 198-210 (2021).

16. Amedi, Amir, Stern, W. M., et al. Shape conveyed by visual-to-auditory sensory substitution activates the lateral occipital complex. *Nat. Neurosci.* **10**, 687–689 (2007).

17. Ricciardi, E., et al. The blind brain: How (lack of) vision shapes the morphological and functional architecture of the human brain. *Exp. Biol. Med.* **239**, 1414-1420 (2014).

18. Handjaras, G. et al. How concepts are encoded in the human brain: A modality independent, category-based cortical organization of semantic knowledge. *NeuroImage* **135**, 232-242 (2016).

19. Wang, X. et al. Two Forms of Knowledge Representations in the Human Brain. *Neuron* **107**, 383-393 (2020).

20. Bedny, M. Evidence from blindness for a cognitively pluripotent cortex. *Trends Cogn. Sci.* **21**, 637-648 (2017).

21. Deen, B. et al. Occipital cortex of blind individuals is functionally coupled with executive control areas of frontal cortex. *J. Cogn. Neurosci.* **27**, 1633-1647 (2015).

22. Waters, D. et al. Fingerspelling, signed language, text and picture processing in deaf native signers: The role of the mid-fusiform gyrus. *NeuroImage* **35,** 1287-1302 (2007).

23. Trettenbrein, P. C. et al. Functional neuroanatomy of language without speech: An ALE meta-analysis of sign language. *Hum. Brain Mapp.* **42** 699-712 (2021).

24. Neville, H. J., et al. Cerebral organization for language in deaf and hearing subjects: Biological constraints and effects of experience. *Proc. Natl. Acad. Sci. U. S. A.* **95**, 922-929 (1998).

25. Lane, C. et al. Reduced left lateralization of language in congenitally blind individuals. *J. Cogn. Neurosci.* **29**, 65-78 (2017).

26.Pant, R., et al. A sensitive period in the neural phenotype of language in blind individuals. *Dev. Cogn. Neurosci.* **41**, 100744 (2020).

27. Friederici, A. D. et al. The role of left inferior frontal and superior temporal cortex in sentence comprehension: Localizing syntactic and semantic processes. *Cereb. Cortex* **13**, 170-177 (2003).

28. Hagoort, P. et al. Integration of Word Meaning and World Knowledge in Language Comprehension. *Science* **304**, 438-441 (2004).

29. Hein, G. et al. Object familiarity and semantic congruency modulate responses in cortical audiovisual integration areas. *J. Neurosci.* **27**, 7881-7887 (2007).

30. Willems, R. M. et al. Differential roles for left inferior frontal and superior temporal cortex in multimodal integration of action and language. *NeuroImage* **47,** 1992-2004 (2009).

31. Kral, A. et al. Higher-order auditory areas in congenital deafness: Top-down interactions and corticocortical decoupling. *Hear. Res.* **343**, 50-63 (2017).

32. Hagoort, P. On Broca, brain, and binding: a new framework. *Trends Cogn. Sci.* **9**, 416-423 (2005).

33. Hasson, U. et al. Intersubject synchronization of cortical activity during natural vision. *Science* **303**, 1634-1640 (2004).

34. Hasson, U. et al. Hierarchical process memory: memory as an integral component of information processing. *Trends Cogn. Sci.* **19**, 304-313 (2015).

35. Anderson, D. R. et al. Cortical activation while watching video montage: An fMRI study. *Media Psychol.* **8**, 7-24 (2006).

36. Fedorenko, E. et al. New method for fMRI investigations of language: defining ROIs functionally in individual subjects. *J. Neurophysiol.* **104**, 1177-1194 (2010).

37. Bedny, M. et al. Language processing in the occipital cortex of congenitally blind adults. *Proc. Natl. Acad. Sci. U. S. A.* **108**, 4429–4434 (2011).

38.Collignon, O. et al. Functional specialization for auditory-spatial processing in the occipital cortex of congenitally blind humans. *Proc. Natl. Acad. Sci. U. S. A.* **108**, 4435–4440 (2011).

39. Watkins, K. E. et al. Early auditory processing in area V5/MT+ of the congenitally blind brain. *J. Neurosci.* **33,** 18242-18246 (2013).

40. Nishimoto, S. et al. Reconstructing visual experiences from brain activity evoked by natural movies. *Curr. Biol.* **21**, 1641-1646 (2011).

41. Huth, A. G. et al. Natural speech reveals the semantic maps that tile human cerebral cortex. *Nature* **532**, 453-458 (2016).

42. de Heer, W. A. et al. The hierarchical cortical organization of human speech processing. *J. Neurosci.* **37**, 6539-6557 (2017).

43. Khosla, M. et al. Cortical response to naturalistic stimuli is largely predictable with deep neural networks. Sci. Adv. **7**, (2021).

44. Hasson, U. et al. A hierarchy of temporal receptive windows in human cortex. *J. Neurosci.* **28**, 2539-2550 (2008).

45.Lerner, Y. et al. Topographic mapping of a hierarchy of temporal receptive windows using a narrated story. *J. Neurosci.* **31**, 2906-2915 (2011).

46. Grall, C., & Finn, E. S. Leveraging the power of media to drive cognition: a media-informed approach to naturalistic neuroscience. *Soc. Cogn. Affect. Neurosci.* **17**, 598-608 (2022).

47. Popham, S. F. et al. Visual and linguistic semantic representations are aligned at the border of human visual cortex. *Nat. Neurosci.* **24**, 1628-1636 (2021).

48.Khaligh-Razavi, S. M. & Kriegeskorte N. Deep supervised, but not unsupervised, models may explain IT cortical representation. *PLoS Comput. Biol.* **10** (2014).

49. Oldfield, R. C. The assessment and analysis of handedness: the Edinburgh inventory. *Neuropsychologia* **9**, 97-113 (1971).

50. Sheehan, P. W. A shortened form of Betts' questionnaire upon mental imagery. *J. Clin. Psychol.* (1967).

51. Marks, D. F. Vividness of visual imagery Questionnaire. *Journal of Mental Imagery* (1973).

52. Andrade, J. et al. Assessing vividness of mental imagery: the Plymouth Sensory Imagery Questionnaire. *Br. J. Psychol.* **105**, 547-563 (2014).

53.DiCarlo, J. J. et al. How does the brain solve visual object recognition?. *Neuron* **73**, 415-434 (2012).

54. Heeger, D. J. et al. Computational models of cortical visual processing. *Proc. Natl. Acad. Sci. U. S. A.* **93** 623-627 (1996).

55. Mikolov, T. et al. Distributed representations of words and phrases and their compositionality. *Adv. Neural Inf. Process Syst.* **26**, (2013).

56. Brown, T. et al. Language models are few-shot learners. *Adv. Neural Inf. Process Syst.* **33**, 1877-1901, (2020).

57. Oliva, A., & Torralba, A. Building the gist of a scene: The role of global image features in recognition. *Prog. Brain Res.* **155**, 23-36 (2006).

58. Lettieri, G. et al. Emotionotopy in the human right temporo-parietal cortex. *Nat. Comm.* **10**(1), 1-13 (2019).

59.Welch, P. The use of fast Fourier transform for the estimation of power spectra: a method based on time averaging over short, modified periodograms. *IEEE Transactions on audio and electroacoustics* **15**, 70-73 (1967).

60. Martinelli, A. et al. Auditory features modelling demonstrates sound envelope representation in striate cortex. Preprint at *bioRxiv* <https://www.biorxiv.org/content/10.1101/2020.04.15.043174v2> (2020).

61. Baroni, M. et al. The WaCky wide web: a collection of very large linguistically processed web-crawled corpora. *Lang. Resour. Eval.* **43**, 209-226 (2009).

62.Cimino, A et al. Multi-task learning in deep neural networks at evalita 2018. *Proceedings of the 6th evaluation campaign of Natural Language Processing and Speech tools for Italian (EVALITA’18)* 86-95 (2018).

63.Radford, A. et al. Language models are unsupervised multitask learners. OpenAI blog **1**, 9 (2019).

64.Schrimpf, M. et al. The neural architecture of language: Integrative modeling converges on predictive processing. *Proc. Natl. Acad. Sci. U. S. A.* **118**(45), (2021).

65.Goldstein, A. et al. Shared computational principles for language processing in humans and deep language models. *Nat. Neurosci.* **25**, 369-380, (2022).

66. Epstein, R. A. Parahippocampal and retrosplenial contributions to human spatial navigation. *Trends Cogn. Sci.* **12**, 388-396 (2008).

67. Kanwisher, N., & Yovel, G. The fusiform face area: a cortical region specialized for the perception of faces. *Philos. Trans. R. Soc. Lond., B, Biol. Sci.* **361**, 2109-2128 (2006).

68. Martin, A. The representation of object concepts in the brain. *Annu. Rev. Psychol.* **58**, 25 (2007).

69. McCandliss, B. D. et al. The visual word form area: Expertise for reading in the fusiform gyrus. *Trends Cogn. Sci.* **7**, 293-299 (2003).

70. Peelen, M. V., & Downing, P. E. Category selectivity in human visual cortex: Beyond visual object recognition. *Neuropsychologia* **105***, 177-183* (2017).

71. Grill-Spector, K., & Weiner, K. S. The functional architecture of the ventral temporal cortex and its role in categorization. *Nat. Rev. Neurosci.* **15,** 536-548 (2014).

72. Kell, A. J. E., & McDermott, J. H. Invariance to background noise as a signature of non-primary auditory cortex. *Nat. Comm.* **10**, 1-11 (2019).

73. Mattioni, S. et al. Categorical representation from sound and sight in the ventral occipito-temporal cortex of sighted and blind. *ELife* **9** (2020).

74. Zheng, S. et al. Conditional random fields as recurrent neural networks. *Proceedings of the IEEE international conference on computer vision* 1529-1537 (2015).

75.Grinsted, A., Moore, J. C. & Jevrejeva, S. Application of the cross wavelet transform and wavelet coherence to geophysical time series. *Nonlinear Process. Geophys.* **11**, 561-566 (2004).

76.Ahlheim, C. & Love, B. C. Estimating the functional dimensionality of neural representations. *Neuroimage* **179**, ​​51-62 (2018).

77.Diedrichsen, J., Wiestler, T. & Ejaz, N. A multivariate method to determine the dimensionality of neural representation from population activity. *Neuroimage* **76**, 225-235 (2013).
