# Supplementary material for: A modality-independent proto-organization of human multisensory areas
Source: Nat Hum Behav. 2023 Jan 16;7(3):397–410. doi: 10.1038/s41562-022-01507-3 (PMC10038796; doi:10.1038/s41562-022-01507-3)
Supplement: Supplementary file 2 — Reporting Summary [file 41562_2022_1507_MOESM2_ESM.pdf]

nature portfolio

Corresponding author(s): Emiliano Ricciardi

Last updated by author(s): Nov 7, 2022

Reporting Summary

Nature Portfolio wishes to improve the reproducibility of the work that we publish. This form provides structure for consistency and transparency in reporting. For further information on Nature Portfolio policies, see our Editorial Policies and the Editorial Policy Checklist.

Statistics

For all statistical analyses, confirm that the following items are present in the figure legend, table legend, main text, or Methods section.

n/a	Confirmed	
☐	☒	The exact sample size (*n*) for each experimental group/condition, given as a discrete number and unit of measurement
☐	☒	A statement on whether measurements were taken from distinct samples or whether the same sample was measured repeatedly
☐	☒	The statistical test(s) used AND whether they are one- or two-sided<br>*Only common tests should be described solely by name; describe more complex techniques in the Methods section.*
☐	☒	A description of all covariates tested
☐	☒	A description of any assumptions or corrections, such as tests of normality and adjustment for multiple comparisons
☐	☒	A full description of the statistical parameters including central tendency (e.g. means) or other basic estimates (e.g. regression coefficient) AND variation (e.g. standard deviation) or associated estimates of uncertainty (e.g. confidence intervals)
☐	☒	For null hypothesis testing, the test statistic (e.g. *F*, *t*, *r*) with confidence intervals, effect sizes, degrees of freedom and *P* value noted<br>*Give P values as exact values whenever suitable.*
☒	☐	For Bayesian analysis, information on the choice of priors and Markov chain Monte Carlo settings
☒	☐	For hierarchical and complex designs, identification of the appropriate level for tests and full reporting of outcomes
☐	☒	Estimates of effect sizes (e.g. Cohen's *d*, Pearson's *r*), indicating how they were calculated

Our web collection on statistics for biologists contains articles on many of the points above.

Software and code

Policy information about availability of computer code

Data collection	Sound mixing was performed with the software from Apple® LogicPro 10.4. The video and audio clips were edited with iMovie software from Apple® (10.1.10) whereas for the creation of subtitles, we rely on the open-source cross-platform Aegisub 3.2.2 (http://www.aegisub.org/). Stimulation was administered through software package Presentation® 16.5 (Neurobehavioral System, Berkeley, CA, USA - http://www.neurobs.com). Brain activity was recorded with Philips 3T Ingenia scanner equipped with a 32-channel head coil.
Data analysis	fMRI data preprocessing and analysis was performed following the standard steps with AFNI_17.1.12 software package and MATLAB R2019b (MathWorks Inc., Natick, MA, USA). Code is available at https://github.com/giacomohandjaras/101_Dalmatians

For manuscripts utilizing custom algorithms or software that are central to the research but not yet described in published literature, software must be made available to editors and reviewers. We strongly encourage code deposition in a community repository (e.g. GitHub). See the Nature Portfolio guidelines for submitting code & software for further information.

Data

Policy information about availability of data

All manuscripts must include a data availability statement. This statement should provide the following information, where applicable:

- Accession codes, unique identifiers, or web links for publicly available datasets
- A description of any restrictions on data availability
- For clinical datasets or third party data, please ensure that the statement adheres to our policy

fMRI data are available on https://osf.io/j8x6h/. Only preprocessed functional data was shared. Raw structural and functional MRI data are available from the corresponding author upon reasonable request to comply with the European General Data Protection Regulation (GDPR). Cortical parcellation was performed using

the HCP Atlas (Glasser et al., 2016, https://www.nature.com/articles/nature18933), projected onto the MNI template (https://identifiers.org/neurovault.collection:1549).

Field-specific reporting

Please select the one below that is the best fit for your research. If you are not sure, read the appropriate sections before making your selection.

☐ Life sciences ☒ Behavioural & social sciences ☐ Ecological, evolutionary & environmental sciences

For a reference copy of the document with all sections, see nature.com/documents/nr-reporting-summary-flat.pdf

Behavioural & social sciences study design

All studies must disclose on these points even when the disclosure is negative.

Study description	3T fMRI study with a naturalistic paradigm (i.e., a movie) administered via three distinct experimental conditions: i) audiovisual, ii) visual-only and iii) auditory-only stimulations. Quantitative, cross-sectional data were acquired.
Research sample	Fifty subjects took part in the study. We enrolled both typically developed (TD) individuals and sensory deprived (SD) subjects, who lack visual or auditory experience since birth. Three samples of TD individuals underwent a different experimental condition consisting in the presentation of one version of the same movie: either i) the full multimodal audiovisual (AV) (n=10, 35±13 years, 8 females), ii) the auditory (A) (n=10, 39±17 years, 7 females) or iii) the visual (V) (n=10, 37±15 years, 5 females) one. SD individuals comprising blind (n=11, mean age 46±14 years, 3 females) and deaf (n=9, mean age 24±4, 5 females) participants were presented with the A and V movie conditions respectively. Congenitally blind and deaf subjects in Europe are extremely rare (<4 out of 10000 newborns for blindness and <2 out of 1000 births for deafness). Moreover, additional eligibility criteria (no metal implants, no medication, no history of neurological or psychiatric disorders, all native Italian speakers) constrained samples recruitment. Sample sizes are comparable to other studies in the field of sensory deprivation. Since our aim was to test the extent to which audiovisual experience is a mandatory prerequisite for the superior temporal cortex to develop and become able to detect shared features between the two sensory streams, we enrolled adult individuals who specifically lack visual or auditory input since birth.
Sampling strategy	The calculation of a suitable sample size was determined by a review of the literature of fMRI studies with naturalistic stimulation and using Intersubject Correlation (ISC) analysis. Results of this procedure are presented in Supplementary Fig.6. Considering the duration of naturalistic stimulation and the subejcts enrolled in the reviewed studies, our sample sizes match with those reported in the literature. Since congenitally deafness and blindness are extremely rare conditions, subject recrtuitment was performed within the two major Italian organizations: the Unione Italiana Ciechi e Ipovedenti (Italian Union of the Blind and Partially Sighted) and the Ente Nazionale Sordi Onlus (Italian Union of the Deaf). Sighted and normal hearing individuals were recruited by word of mouth.
Data collection	Structural and functional data acquisition were performed on a single scanning day during the presentation of the narrative. Brain activity was recorded with Philips 3T Ingenia scanner equipped with a 32-channel head coil. Functional images were acquired using gradient recall echo planar imaging (GRE-EPI). In the same session, three-dimensional high-resolution anatomical image of the brain was also acquired using a magnetization-prepared rapid gradient echo (MPRAGE). Audio and visual stimulation were delivered through MR-compatible LCD goggles and headphones (VisualStim Resonance Technology, video resolution 800x600 at 60 Hz, visual field 30° × 22°, 5, audio 30 dB noise-attenuation, 40 Hz to 40 kHz frequency response). Both goggles and headphones were prescribed irrespectively of the experimental condition and group membership, meaning that each subject wore both devices. The video and audio clips were administered through software package Presentation® 16.5 (Neurobehavioral System, Berkeley, CA, USA - http://www.neurobs.com). Congenitally deaf individuals were accompanied by a sign language interpreter who explained the task and assisted them. Researchers were not blind to the experimental conditions.
Timing	Data acquisition started in December 2018 and ended in October 2019.
Data exclusions	Two blind subjects were removed from the fMRI analysis for excessive head movement (final sample, n=9, mean age 44±14 years, 3 females).
Non-participation	No participants dropped out or declined the participation.
Randomization	TD participants were randomly allocated to the audiovisual, visual-only or auditory-only conditions.

Reporting for specific materials, systems and methods

We require information from authors about some types of materials, experimental systems and methods used in many studies. Here, indicate whether each material, system or method listed is relevant to your study. If you are not sure if a list item applies to your research, read the appropriate section before selecting a response.

Materials & experimental systems

n/a	Involved in the study
☒	☐ Antibodies
☒	☐ Eukaryotic cell lines
☒	☐ Palaeontology and archaeology
☒	☐ Animals and other organisms
☐	☒ Human research participants
☒	☐ Clinical data
☒	☐ Dual use research of concern

Methods

n/a	Involved in the study
☒	☐ ChIP-seq
☒	☐ Flow cytometry
☐	☒ MRI-based neuroimaging

Human research participants

Policy information about studies involving human research participants

Population characteristics	Causes of congenital blindness were heterogenous: retinopathy of prematurity, retinitis pigmentosa, optic nerve atrophy, Leber congenital amaurosis, retinal detachment, bilateral retinoblastoma. All blind participants did not have any residual light perception. They all learned Braille reading at the age of six. All deaf participants reported to suffer from a hereditary form of congenital deafness but one subject who underwent sensorineural hearing loss in the first months of life due to high fever. They reported to have learned Italian Sign Language (LIS) as the first language. Eight out of nine congenitally deaf participants used hearing aids during childhood, while one of them still utilized the device in his life at the moment of the study.<br>For additional demographic characteristics (e.g., age, sex) of the samples please see above.
Recruitment	Due to the extremely low prevalence of congenitally deaf and blind individuals in the population, sensory deprived participants were recruited using the snowball sampling procedure. Typically developed individuals were recruited within the students of the Psychology program at the University of Turin as well as by word of mount among their acquaintances to match age and sex characteristics.
Ethics oversight	The study was approved by the Ethical Committee of the University of Turin (protocol n. 195874, 05/29/19) and conforms to the Declaration of Helsinki.

Note that full information on the approval of the study protocol must also be provided in the manuscript.

Magnetic resonance imaging

Experimental design

Design type	Naturalistic Stimulation
Design specifications	Movie presentation was split in 6 runs of about 8 minutes duration each. Afterwards, the experimental paradigms involved an additional (8 minutes) run consisting in a scrambled version of the narrative, as control condition for the processing of the story semantic. Runs were presented consecutively and were separated by brief intervals in which the experimenter communicated with the subjects to check their compliance and ascertain everything was good during the scanning. No specific task was provided to the participants that were just told to “follow the plot and enjoy the movie”.
Behavioral performance measures	Before starting the scanning session, participants were asked to rate their general knowledge of the movie plot on a Likert scale ranging from 1 (not at all) to 5 (very well), and at the end of the experiment, an ad hoc two-alternative forced choice questionnaire about the content of the story was administered at the end of the experiment to assess subject engagement and compliance.

Acquisition

Imaging type(s)	Structural and functional MRI data.
Field strength	3T
Sequence & imaging parameters	Functional images: GRE-EPI; TR = 2000 ms; TE = 30 ms; FA = 75°; FOV = 240 mm; matrix size (in plane resolution) = 80 × 80; slice thickness = 3 mm; voxel size =3x3x3 mm; 38 sequential axial ascending slices. Anatomical image: MPRAGE; TR =7 ms; TE = 3.2 ms; FA = 9°; FOV= 224, matrix size = 224 x 224; slice thickness = 1mm; voxel size = 1x1x1 mm; 156 sagittal slices).
Area of acquisition	whole brain scans

Diffusion MRI ☐ Used ☒ Not used

Preprocessing

Preprocessing software	AFNI_17.1.12 and MATLAB R2019b (MathWorks Inc., Natick, MA, USA) software packages were used.

Normalization	single subject fMRI volumes were nonlinearly (3dQWarp) registered to the MNI-192 standard space.
Normalization template	MNI-192 standard space.
Noise and artifact removal	we removed scanner-related noise correcting the data by spike removal (3dDespike). Head motion was corrected using as base the first run (3dvolreg). A multiple regression analysis was performed (3dDeconvolve) to remove signals related to head motion parameters, and movement spike regressors (frame wise displacement above 0.3). Signal trends were removed using a Savitzky-Golay filter in Matlab.
Volume censoring	no volumes were excluded from the analysis.

Statistical modeling & inference

Model type and settings	Inter-Subject Correlation (ISC) during a naturalistic stimulation, considering subject pairings as a random effect. Statistical significance of the inter-subject synchronization was evaluated throughout permutation tests by shuffling fMRI time series preserving low- and high-frequency fluctuations. A model-mediated version of ISC was developed to measure the impact on subjects' synchronization of multiple sets of computational features. Statistical significance of the model-mediated ISC was obtained using null models generated by means of the IAAFT (Iterative Amplitude Adjusted Fourier Transform) algorithm, to preserve spectral as well as distributional properties of the original features.
Effect(s) tested	ISC during multimodal stimulation (average Pearson correlation coefficient of every possible subjects' pair in each voxel independently). ISC across modality, in which we measured the synchronization between subjects exposed to the auditory stimulation only and those administered with the visual stimulation only. ISC across modality was performed in typically developed (TD) individuals and sensory deprived (SD) subjects. We tested in each condition whether ISC was significant greater than zero (one-tailed, non-parametric test), and the differences of ISC magnitude between TD and SD participants (two-tailed, Wilcoxon rank sum test). As concerns the model-mediated ISC, we tested whether each set of computational features was significantly able to reduce the across-modality synchronization in TD and SD participants (one-tailed non-parametric test).

Specify type of analysis: ☐ Whole brain ☐ ROI-based ☒ Both

Anatomical location(s): *Describe how anatomical locations were determined (e.g. specify whether automated labeling algorithms or probabilistic atlases were used).*

Statistic type for inference (See Eklund et al. 2016)	voxel-wise
Correction	FWE, Bonferroni.

Models & analysis

n/a	Involved in the study
☒	☐ Functional and/or effective connectivity
☒	☐ Graph analysis
☒	☐ Multivariate modeling or predictive analysis
